# Intranasal rapamycin ameliorates Alzheimer-like cognitive decline in a mouse model of Down syndrome

**DOI:** 10.1186/s40035-018-0133-9

**Published:** 2018-11-06

**Authors:** Antonella Tramutola, Chiara Lanzillotta, Eugenio Barone, Andrea Arena, Ilaria Zuliani, Luciana Mosca, Carla Blarzino, D. Allan Butterfield, Marzia Perluigi, Fabio Di Domenico

**Affiliations:** 1 Department of Biochemical Sciences, Sapienza University of Rome, P.le Aldo Moro 5, 00185 Rome, Italy; 2 Universidad Autònoma de Chile, Instituto de Ciencias Biomédicas, Facultad de alud, Avenida Pedro de Valdivia 425, Providencia, Santiago, Chile; 3 Department of Chemistry and Sanders-Brown Center on Aging, University of Kentucky, Lexington, KY 40506-0055 USA

**Keywords:** mTOR, Autophagy, Rapamycin, Down syndrome, Alzheimer disease, APP, Tau, Oxidative stress

## Abstract

**Background:**

Down syndrome (DS) individuals, by the age of 40s, are at increased risk to develop Alzheimer-like dementia, with deposition in brain of senile plaques and neurofibrillary tangles. Our laboratory recently demonstrated the disturbance of PI3K/AKT/mTOR axis in DS brain, prior and after the development of Alzheimer Disease (AD). The aberrant modulation of the mTOR signalling in DS and AD age-related cognitive decline affects crucial neuronal pathways, including insulin signaling and autophagy, involved in pathology onset and progression. Within this context, the therapeutic use of mTOR-inhibitors may prevent/attenuate the neurodegenerative phenomena. By our work we aimed to rescue mTOR signalling in DS mice by a novel rapamycin intranasal administration protocol (InRapa) that maximizes brain delivery and reduce systemic side effects.

**Methods:**

Ts65Dn mice were administered with InRapa for 12 weeks, starting at 6 months of age demonstrating, at the end of the treatment by radial arms maze and novel object recognition testing, rescued cognition.

**Results:**

The analysis of mTOR signalling, after InRapa, demonstrated in Ts65Dn mice hippocampus the inhibition of mTOR (reduced to physiological levels), which led, through the rescue of autophagy and insulin signalling, to reduced APP levels, APP processing and APP metabolites production, as well as, to reduced tau hyperphosphorylation. In addition, a reduction of oxidative stress markers was also observed.

**Discussion:**

These findings demonstrate that chronic InRapa administration is able to exert a neuroprotective effect on Ts65Dn hippocampus by reducing AD pathological hallmarks and by restoring protein homeostasis, thus ultimately resulting in improved cognition. Results are discussed in term of a potential novel targeted therapeutic approach to reduce cognitive decline and AD-like neuropathology in DS individuals.

**Electronic supplementary material:**

The online version of this article (10.1186/s40035-018-0133-9) contains supplementary material, which is available to authorized users.

## Background

Down syndrome (DS) is the most common genetic cause of intellectual disability due to total or partial triplication of chromosome 21 (trisomy 21) [1]. The increased risk to develop Alzheimer-like dementia in DS individuals is becoming a key issue to manage the extension of the lifespan of DS population. Indeed, if from one side the improved quality of life and the longer life expectancy are significant achievements of both social and medical care, the overall increase of mean age of DS individuals is associated with an elevated risk to develop age-associated disorders, among which Alzheimer disease (AD) [2]. The neuropathological conditions of DS subjects are complex and involve: deposition of senile plaques and neurofibrillary tangles, dysfunctional mitochondria, defective neurogenesis, increased oxidative stress and altered proteostasis [3]. Approximately two-thirds of individuals with DS develop dementia and brain pathological hallmarks in their 50s, but severity varies significantly among DS population [1]. The triplication of amyloid precursor protein (APP) is considered the major pathological event in both AD and DS with AD, but it is likely that several other triplicated genes contribute to the neurodegenerative process [2, 4–6]. Our previous studies investigated the molecular mechanisms responsible for early onset of AD in DS, focusing attention on the mechanisms that lead to the impairment of protein quality control (PQC) pathways, including the ubiquitin proteasome system (UPS) and autophagy [7, 8]. We showed, in the frontal cortex from DS individuals before and after development of AD neuropathology, that key components of the PQC are irreversibly oxidatively modified resulting in aberrant protein functionality [7, 9]. In agreement, we observed, in young DS subjects, the early accumulation of polyubiquitinylated proteins before the appearance of AD symptoms [10, 11]. These data suggest that impairment of protein degradative system may play a crucial role in the early accumulation of amyloid beta (Aβ) and tau toxic protein aggregates, thus accelerating the neurodegenerative process. Collecting studies suggest that, in AD brain and animal models thereof, the reduced autophagy is strongly associated with the hyperactivation of the PI3K/AKT/mTOR axis, leading to the accumulation of protein aggregates [12, 13]. In the central nervous system (CNS), mTOR and its downstream signalling pathways are involved in synaptic plasticity, memory retention, neuroendocrine regulation, puberty, and neuronal recovery [14–16]. Dysregulation of the mTOR pathway is emerging as a leitmotif in a large number of human diseases, including cancer, metabolic syndromes, and neurological disorders. In the last decade, great attention has been dedicated to the role of mTOR in the development of AD. mTOR hyperactivity is observed in AD brains from human and mouse models, and strong evidence demonstrated that alterations of mTOR may be one of the leading events contributing to the formation of toxic aggregates during AD pathology [17–20]. Recent studies from our laboratory and others employing specimens from DS individuals and DS mouse models confirmed that aberrant mTOR signalling is an early degenerating event in the brain that contributes to acceleration of Aβ and tau deposition and to the development of AD-like cognitive decline [7, 9, 13, 21, 22]. In particular, we investigated the status of the PI3K/AKT/mTOR pathway in the frontal cortex from DS autopsy cases without AD neuropathology (typically under the age of 40 years) and DS with AD neuropathology [13]. Our results showed the hyperactivation of the PI3K/AKT/mTOR axis in the brains of subjects with DS with or without AD pathology in comparison to healthy individuals. These data were associated with decreased autophagosome formation and increased levels of Aβ and p-tau.

These findings represent a strong rationale to test therapeutic strategies aimed to restore the functionality of PQC or prevent its gradual loss. Among drug candidates, mTOR inhibitors led to enormous interest as potential AD-modifying agents [20, 23–28], thus representing an appealing potential approach against neurodegeneration. Evidence showing the positive effects on memory of orally administered rapamycin demonstrated the concomitant reduction, of AD pathological markers, including Aβ and tau levels, in Tg mouse models of AD [16, 19, 27, 29–32]. In the present work, we employed a novel therapeutic strategy using rapamycin, a selective inhibitor of mTOR, delivered by intranasal route in order to avoid peripheral accumulation. Our treatment supports the pathological role of aberrant mTOR/autophagy axis in DS mice and propose/confirm mTOR as a valuable target to prevent/slow the development of AD-related cognitive decline in DS as well as in the general population.

## Methods

### Mouse colony

Ts65Dn (B6EiC3Sn a/A-Ts(1716)65Dn/J), a well-established mouse model of DS, carries a reciprocal translocation that is trisomic for approximately 104 genes (56%) on Mmu16, from Mrpl39 to the distal telomere, with homologues on HSA21. Mice were generated by repeatedly backcrossing Ts65Dn trisomic females with (B6EiC3SnF1/J) F1 hybrid males. The parental generations were purchased from Jackson Laboratories (Bar Harbour, ME, USA). These breeding pairs produce litters containing both trisomic (Ts65Dn) and euploid (Eu) offsprings. The pups were genotyped to determine trisomy using standard PCR, as described by Reinoldth [33]. In addition, the recessive retinal degeneration 1 mutation (Pdebrd1), which segregates in the colony and results in blindness in homozygotes, was analyzed for all trisomic animals used in the present study by standard PCR. Animals expressing the mutant gene were excluded from the behavioral studies. Mice were housed in clear Plexiglas cages (20 × 22 × 20 cm) under standard laboratory conditions with a temperature of 22 ± 2 °C and 70% humidity, a 12-h light/dark cycle and free access to food and water. Littermates were spliced among age groups to avoid littermates/dam-specific effects. Mice characteristics are reported in Table 1. Mice were sacrificed by cervical dislocation. Trunk blood was collected from the site where the animal was decapitated. Animals were perfused with saline and one brain hemisphere was dissected for immunochemical analysis while the other brain hemisphere was fixed and collected for immunofluorescence staining. All the experiments were performed in strict compliance with the Italian National Laws (DL 116/92), the European Communities Council Directives (86/609/EEC). Experimental protocol was approved by Italian Ministry of Health authorization n° 1183/2016-PR. All efforts were made to minimize the number of animals used in the study and their suffering. All samples were flash-frozen and stored at − 80 °C until utilization.

**Table 1:** Mouse samples characteristics and experimental use

Treatment	Genotyping	n	Gender (m/f)	Weight (AVG ± SD)	Pde6b	Experimental Use (n)			
						Behavioral Tests	WB	IF	Q-PCR
Vehicle	Eu	10	6/4	31.5 ± 7.2	0	10	8	4	6
	Ts65Dn	10	5/5	25.7 ± 3.2	0	10	8	4	6
InRapa	Eu	10	6/4	31.8 ± 6.8	0	10	8	4	6
	Ts65Dn	10	6/4	28.9 ± 5.6	0	10	8	4	6

### InRapa treatment

**Fig. 1:**
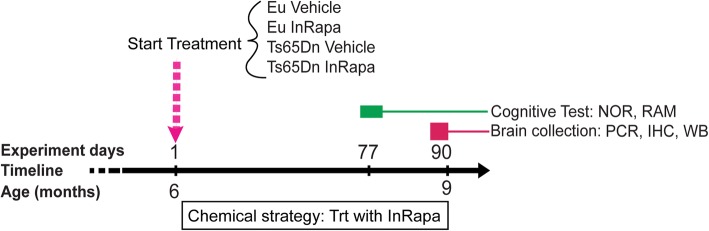
Schematic of InRapa treatment. 6-month old Ts65Dn mice and euploid (Eu) were administered with intranasal rapamycin (InRapa; Rapamune, Pfizer) 1 μg/mouse and Vehicle (Veh; saline with 1% DMSO) for 90 days total. At day 77 cognitive tests (NOR and RAM) has been initiated while at day 90 mice has been sacrificed to perform PCR, IHC and WB on collected brain samples

6-month old Ts65Dn mice and euploid were administered intranasal rapamycin (InRapa; Rapamune, Pfizer, New York, NY, USA) and Vehicle (Veh; saline with 1% DMSO) for 12 weeks. Mice were divided in 4 experimental groups euploid and Ts65Dn treated with vehicle or rapamycin (*n* = 10 per group). The treatment was conducted 3 times per week, with a dose of 0.1 μg/μl of rapamycin solution or vehicle (Fig. 1) in 10 μl (1 μg/mouse). The treatment was well tolerated and no change in body weight or in the consumption of drinking water was observed. The rapamycin dose was chosen from a dose-response pilot study performed before the treatment. In the dose–response treatment the animals were divided in three groups (*n* = 6 per group) and treated with 0.01, 0.05, 0.1 and 0.2 μg/μl of rapamycin. Our data demonstrated that the dose of rapamycin administered during the treatment, 0.1 μg/μl, was able to partially inhibit mTOR (Ser2448) phosphorylation in mice hippocampus and frontal cortex (see Additional file 1). Rapamycin distribution in brain and plasma was investigated before treatment by UPLC-MS analysis. Briefly 6 euploids mice were treated by single intranasal delivery of rapamycin, with the dose of 1 μg/mouse (0.05 mg/Kg/mouse), or by single intraperitoneal injection (i.p.) with the dose of 50 μg/mouse (2.5 mg/kg/mouse). 4 h after the treatment brain and blood were collect for analysis. In particular, for plasma isolation blood was collected in presence of EDTA and then centrifuged at 3000×g for 15 min at 4 °C.

### Ultra-performance/pressure liquid chromatography- mass spectrometry (UPLC-MS) analysis

For the quantification of rapamycin in brain and plasma, a UPLC-MS analysis method was utilized. Collected biological specimens were prepared as follows. Brain samples (≈100 mg) were homogenized by 20 strokes of a Wheaton tissue homogenizer using 200 μl of a lysis buffer (30 mM Tris-HCl, 0.1 M NaCl, pH 7.4). Further homogenization was obtained through sonication of the samples for 10 s 3-times in ice. Homogenized brain and isolated plasma (100 μl) samples were then purified using an Ostro™ Pass-through Sample Preparation Plate (Waters) to remove proteins and phospholipids, by following the instructions provided by the manufacturer. The samples were finally dried under vacuum at low temperature. The residue was resuspended in 50 μl of water/acetonitrile (20:80) and 40 μl were injected onto the instrument. Chromatographic separation was performed on a Waters Acquity H-Class UPLC system (Waters, Milford, MA, USA), including a quaternary solvent manager (QSM), a sample manager with flow through needle system (FTN), a photodiode array detector and a single-quadruple mass detector with electrospray ionization source (ACQUITY QDa). The column was a Zorbax Eclipse-Plus C8 (4.6 × 50 mm, 1.8 μm particle size). The mobile phase was composed of a 5 mM ammonium formate aqueous solution (Solvent A) and 0.1% formic acid in methanol (Solvent B). A gradient elution program was performed starting with 30% solvent A and 70% solvent B for 3 min, up to 100% B in 3 min, followed by 2 min at 100% B. The column was re-equilibrated to 30% A/70% B for 4 min. In these conditions rapamycin has a retention time of 5.4 min. Mass spectrometric detection was set in the positive electrospray ionization mode using nitrogen as nebulizer gas. Analyzes were performed in Total Ion Current (TIC) mode in the mass range 400–1000 m/z. Capillary voltage was 0.8 kV, cone voltage 30 V, ion source temperature 120 °C and probe temperature 600 °C. QDa analysis detected the presence of rapamycin adducts with potassium, sodium and ammonium ions ([M + K]^+^*m/z* = 952.45, [M + Na]^+^*m/z* = 936.45, [M + NH_4_]^+^*m/z* = 931.45). The quantification of rapamycin adducts was performed by automatic peak area integration using a dedicated software (Empower3). A calibration curve was plotted using different amounts of rapamycin (0.5 pmoles to 200 pmoles), treated with the same procedure used for the samples. The curves (11 data points in duplicate) were linear with an R^2^ value of ≈ 1.00.

### Novel object recognition (NOR)

The Novel Object Recognition (NOR) task is used to evaluate cognition, particularly recognition memory, in rodent models of CNS disorders. All experimental groups (Eu Veh, Ts65Dn Veh, Eu InRapa and Ts65Dn InRapa) were involved in the test procedures. This test is based on the spontaneous tendency of rodents to spend more time exploring a novel object than a familiar one. The task procedure consists of three phases: habituation, familiarization, and test phase. In the habituation phase at 1st day, each animal is allowed 10 min to freely exploring the open-field arena (50 cm deep × 30 cm widths × 30 cm height) in the absence of objects. During the familiarization phase on the 2nd day, a single animal is placed in the open-field arena containing two identical objects (two balls), for 10 min. To prevent coercion to explore the objects, rodents are released against the center of the opposite wall with its back to the objects. The experimental context is not drastically different during the familiarization and the test phase. In the test phase after 24 h, the animal is returned to the open-field arena with two objects, one is the familiar object and the other is novel (ball + plastic brick) [34, 35]. The discrimination index and preference index percentage are recorded. Discrimination index (DI), allows discrimination between the novel (TN) and familiar (TF) objects [DI = (TN − TF)/(TN + TF)]. The preference index (PI) is a ratio of the amount of time spent exploring any one of the two objects in training phase (A, B) or the novel one in test phase (C) over the total time spent exploring both objects, i.e., A, B or C/(A, B + C) × 100 (%) in the test phase. Therefore, a preference index above 50% indicates novel object preference, below 50% familiar object preference, and 50% no preference [35].

### Radial arms maze (RAM)

The Radial Arms Maze is composed of a central octagonal platform with eight arms extending from it like the spokes of a wheel [36]. All animals were familiarized with the maze for 3 days before training (Eu Veh, Ts65Dn Veh, Eu InRapa and Ts65Dn InRapa; *n* = 10). On these 3 days, they were placed in the maze for 10 min and could eat food rewards that were scattered throughout the maze. In our protocol, the version of the task was an alternated- baited maze procedure, where mice had to learn to visit only all the baited arms. Mice were given daily training sessions (one trial per session) over a 9-day period and day 10th was considered a Test Day. A daily training session started with the animal placed in the central area, once the mouse explored the baited arm and came back into the central area, the trial was ended. For all groups, a trial ended when one of the following conditions was reached: (i) the animal visited all baited arms, or (ii) the trial lasted for more than 10 min. The maze was cleaned with absorbing paper between each animal to minimize the olfactory intra-maze cues. We evaluated the total number of working memory errors (WME), reference memory errors (RME) and latency to finish a trial made by the animals with respect to the training sessions. Distance travelled between groups have been recorded and analyzed showing no significant differences (Additional file 2).

### RNA extraction and quantitative real-time RT-PCR

RNA was extracted from the frozen hippocampus in the all groups (*n* = 6/group) using Tissue Total RNA Kit according to manufacturer’s instructions (Fisher Molecular biology, Rome, Italy). RNA was quantified using the Biospec Nano spectrophotometer (Shimadzu, Columbia, MD, USA), and RNA was reverse transcribed using the cDNA High Capacity kit (Applied Biosystems, Foster City, CA, USA), including reverse transcriptase, random primers and buffer according to manufacturer’s instructions. The cDNA was produced through a series of heating and annealing cycles in the MultiGene OPTIMAX 96-well Thermocycler (LabNet International, Edison, NJ, USA). Real time PCR (Q-PCR) using the following cycling conditions: 35 cycles of denaturation at 95 °C for 20 s; annealing and extension at 60 °C for 20 s, using the SensiFAST™ SYBR® No-ROX Kit (Bioline, London, UK). PCR reactions were carried out in a 20 μl reaction volume in a CFX Connect Real Time PCR machine (Bio-Rad Laboratories, Hercules, CA, USA). Primers used for the evaluation of gene expression are reported in Table 2. Relative mRNA concentrations were calculated from the take-off point of reactions (threshold cycle, Ct) using the comparative quantification method performed by Bio-Rad software and based upon the ∆∆Ct method. Ct values for GAPDH expression served as a normalizing signal [37].

### Sample preparation for Western blot and immunofluorescence

Brain tissues of Ts65Dn and euploid mice (*n* = 8 per group) after treatment were sagittally divided in right and left hemispheres. The right portion was used for Immunofluorescence studies and the left portion was used for molecular biology studies. For western blot and slot blot, the left-hippocampus were thawed in RIPA buffer (pH 7.4) containing 50 mM Tris-HCl (pH 7.4), 150 mM NaCl, 1% NP-40, 0.25% sodium deoxycholate,1 mM EDTA, 0,1% SDS, 1 mM PMSF, 1 mM NaF and 1 mM Na_3_VO_4_. Brains were homogenized by 20 strokes of a Wheaton tissue homogenizer. All the samples homogenate was centrifuged at 14,000×g for 10 min to remove cellular debris. The supernatant was extracted to determine the total protein concentration by the BCA method (Pierce, Rockford, IL, USA).

### Western blot

For Western blot, 30 μg of proteins were prepared by adding in 2X Laemmli Buffer (Bio-Rad Laboratories, Hercules, CA, USA). The sample was heated at 100 °C for 10 min. Electrophoresis was performed on the samples using a Criterion TGX Stain-Free 4–15% 18-well gel in a Criterion large format electrophoresis cell (Bio-Rad Laboratories, Hercules, CA, USA) in TGS Running Buffer (Bio-Rad Laboratories, Hercules, CA, USA), for 60 min at 100 V. Immediately after electrophoresis, the gel was placed on a Chemi/UV/Stain-Free tray and then placed into a ChemiDoc MP imaging System (Bio-Rad Laboratories, Hercules, CA, USA) and UV-activated based on the appropriate settings with Image Lab Software (Bio-Rad Laboratories, Hercules, CA, USA). For gels that would be later used in blotting, the software-selected activation time was 45 s. Following electrophoresis and gel imaging, the proteins were transferred via the TransBlot Turbo semi-dry blotting apparatus (Bio-Rad Laboratories, Hercules, CA, USA) onto a nitrocellulose membrane. After transfer, the blot was imaged using the ChemiDoc MP imaging system using the Stain-Free Blot settings. This total protein signal was used as the basis for total protein normalization. Membranes were blocked with 3% of bovine serum albumin (SERVA Electrophoresis GmbH, Heidelberg, Germany) and incubated over night at 4 °C with primary antibody. An additional table shows the antibody details (see Additional file 3). All the membranes were incubated for 1 h at room temperature with secondary antibody horseradish peroxidase-conjugated anti-rabbit, anti-mouse or anti-goat IgG (1:5000, Sigma–Aldrich, St Louis, MO, USA). The blot was then imaged via the ChemiDoc MP imaging system using the Chemiluminescence settings. Subsequent determination of relative abundance via total protein normalization was calculated using Image Lab 6.0 software (Bio-Rad Laboratories, Hercules, CA, USA).

### Slot blot

For the analysis of total 3-nitrotyrosine (3-NT) and 4-hydroxy-2-nonenal (HNE)-bound protein levels, 10 μl of hippocampus homogenate were incubated with 10 μl of Laemmli buffer containing 0.125 M Tris base pH 6.8, 4% (*v*/v) SDS, and 20% (v/v) glycerol. The resulting samples (250 ng per well) were loaded onto a nitrocellulose membrane with a slot-blot apparatus under vacuum pressure. The membrane was blocked for 2 h with a solution of 3% (*w*/*v*) bovine serum albumin in PBS containing 0.01% (w/v) sodium azide and 0.2% (v/v) Tween 20 and incubated respectively with primary antibodies anti-HNE (Alpha diagnostic, San Antonio, TX, USA) and anti-3NT (Santa Cruz Biotechnology, Dallas, TX, USA) for 2 h at RT. Membranes were washed and incubated with anti-rabbit or mouse IgG alkaline phosphatase secondary antibodies (Sigma-Aldrich, St Louis, MO, USA) for 1 h at room temperature. The membrane was developed with Sigma fast tablets (5-bromo-4-chloro-3-indolyl phosphate/nitroblue tetrazolium substrate [BCIP/NBT substrate], Sigma-Aldrich, St Louis, MO, USA). Membranes were dried and the image was acquired using ChemiDoc XP image system and analyzed using Image Lab software (Bio-Rad Laboratories, Hercules, CA, USA).

### Immunofluorescence

Brains were removed and immersed in 4% paraformaldehyde for 24 h at 4 °C. Fixed brains were cryoprotected in successive 48 h with a solution of 20% sucrose and 0.02% NaN_3_ at 4 °C. Brains were frozen on a temperature-controlled freezing stage, coronal sectioned (20 μm) on a sliding cryostat (Leica Biosystems, Wetzlar, Germany), and stored in a solution of PBS containing 0.02% NaN_3_ at 4 °C. Brain sections were mounted on glass slide. Once dried, sections were blocked with a solution containing 10% normal goat serum, 0.02% NaN_3_, and 0.2% Triton X-100 in TBS. Slides were then incubated overnight at 4 °C with following antibodies: p(Ser 2448)-mTOR (mouse 1:500), p(Ser416)-tau (rabbit 1:500), amyloid-β (B-4) (rabbit 1:500) (Bio-Rad Laboratories, Hercules, CA, USA; Santa Cruz Biotechnology, Dallas, TX, USA). Slides were then washed with TBS and then incubated with Alexa Fluor -488 nm and -594 nm secondary antibodies (Invitrogen Corporation, Carlsbad, CA, USA) at 1:1500 for 2 h at room temperature. Slides were then washed again and incubated with DAPI (1:10.000). One slide per group was stained omitting primary antibodies to establish nonspecific background signal. Cover slips were placed using a drop of Fluorimount (Sigma-Aldrich, St Louis, MO, USA).

All slides were imaged using Zeiss AXio (Carl Zeiss, Oberkochen, Germany). All immunolabeling acquisition intensities, field sizes, and microscopy settings were kept consistent across all images. Images were analyzed using ImageJ. Image montages for Figures were collated in Illustrator and Photoshop Cs6 (Adobe System, San Josè, CA, USA) software programs and were based upon cells that most closely approximated the group means.

### Experimental size and statistical analysis

Behavioural tests (NOR and RAM) were performed using 10 mice per group (Ts65Dn Veh and Rapa; Eu Veh and Rapa). Q-PCR was achieved one time with cDNA from 6 mice per group (Ts65Dn Veh and Rapa; Eu Veh and Rapa). Each immunoblot experiment was performed at least three times using 8 samples per group. Immunohistochemistry analyzes were performed using at least 10 sections per brain of 4 mice per group. Details on sample size are summarized in Table 1). All statistical analyses were performed using a non-parametric one-way ANOVA with post hoc Bonferroni t-test. Further to determine how our data are affected by genotype (DS), treatment (InRapa) and their interaction we accomplished a two-way ANOVA analysis (data are reported in a table as Additional file 4). Data are expressed as mean ± SD per group. All statistical analyzes were performed using Graph Pad Prism 7.0 software (GraphPad, La Jolla, CA, USA).

## Results

### Intranasal delivery reduces peripheral rapamycin concentration

To test the advantage of intranasal delivery in comparison to intraperitoneal (i.p.) injection, we treated mice once by the intranasal route, with the dose of 1 μg/mouse (0.05 mg/Kg/mouse), and by i.p. 50 μg/mouse (2.5 mg/kg/mouse) as previously reported [20]. To note, InRapa dose is about 50-times lower than i.p injection dose. After 4 h of treatment we sacrificed mice and analyzed brain and plasma, from InRapa and i.p. treated animals, by UPLC-MS, to evaluate rapamycin distribution. Our analysis demonstrates that InRapa treated mice showed a brain concentration of rapamycin of 5.0 ± 1.0 ng/g after 4 h, while the plasma concentration was 6.7 ± 1.3 ng/ml (see Additional file 5). In contrast, animals treated by i.p. injection showed a rapamycin brain concentration of 11.1 ± 1.7 ng/g and a plasma concentration of 890.5 ± 98.1 ng/ml (see Additional file 5). Collectively, our data demonstrate that rapamycin delivered by intranasal route reached a therapeutic brain concentration comparable to that obtained by i.p. injection but with an extremely lower distribution at plasma level. Therefore, these results, coupled with the analysis by WB of mTOR inhibition in liver and heart tissue, which showed no changes between Ts65Dn rapamycin and vehicle treated groups (see Additional file 6), suggest that InRapa delivery might not yield consistent side effects in peripheral organs.

### InRapa improves cognitive performances in Ts65Dn mice

**Fig. 2:**
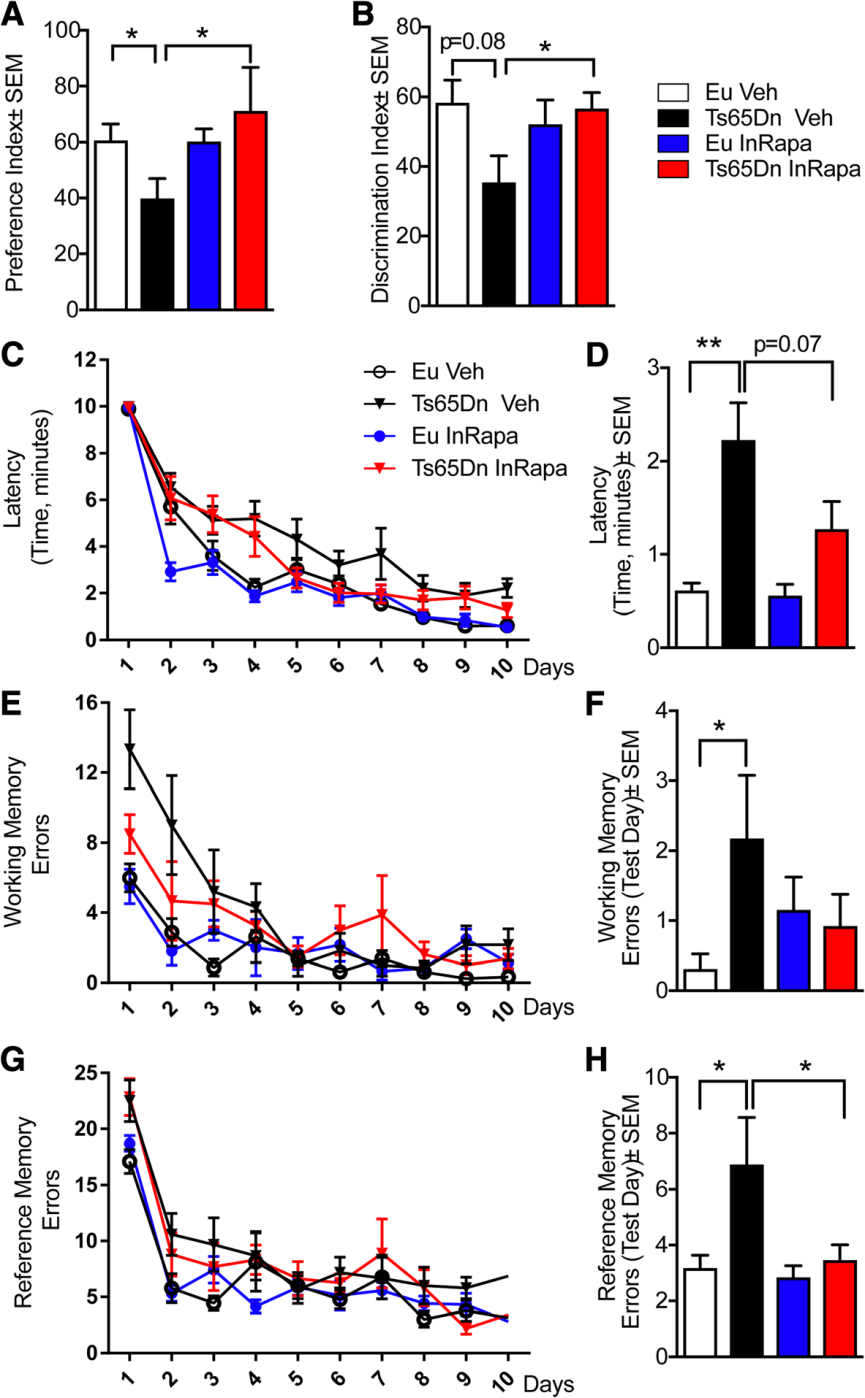
InRapa improves cognitive performances in TS65Dn mice. Panel **a** and **b** represent data of the novel object recognition test. Values shown in the bar graph are in (**a**) Preference index and in (**b**) Discrimination index (data presented are mean ± SEM *n* = 10/ group). Statistical significance was determined using one–way ANOVA and post hoc Bonferroni t-test (*p < 0.05, **p < 0.01). Panels from **c** to **h** represent Radial Arm Maze (RAM) results for our treatment groups of treatment. The red and black triangles are data from Ts65Dn mice treated with InRapa and Veh solution. The blue filled circles are data from Eu mice treated with InRapa and the empty circle are data from Eu mice treated with Veh solution. Panel **c** represents the latency of the mice on each trial (one trial per day). Panel **d** represents bar diagram showing latency during the test day (day 10). Panel **e** represents the working memory errors committed by mice on each trial (one trial per day). Panel **f** represents bar diagram showing working memory error during the test day (day 10). Panel **g** represents the reference memory errors committed by mice on each trial (one trial per day). Panel **h** represents bar diagram showing reference memory error during the test day (day 10) values shown in the bar graph are the mean of 10 samples per each group

To evaluate the effects of InRapa treatment on cognitive performances mice were subjected before the end of the treatment to hippocampal-based tasks, novel object recognition test (NOR) and eight-arms radial arms maze test (RAM), to test spatial learning and working memory, at first, to assess memory status differences between treated (InRapa) and untreated (Veh) animals, we performed the NOR tests. Our data show that Eu animals, both vehicle and rapamycin treated groups, demonstrate a PI above 50%, while Ts65Dn mice treated with vehicle exhibit a PI slightly below 50% as result of hippocampal alterations. Interestingly, InRapa treatment is able to recover PI in Ts65Dn mice (increased about 70%; *p* = 0.04) and present a significant difference with Ts65Dn Veh mice, suggesting a recovery of hippocampal functions after InRapa administration (Fig. 2a). In addition, the impairment of cognition in Ts65Dn Veh is demonstrated by the significant reduction of DI (20%; *p* = 0.08) (Fig. 2b) when compared to Eu Veh, while Ts65Dn InRapa group, demonstrate increased DI in comparison with Ts65Dn Veh group (about 20%, *p* = 0.019). The analysis of data by two-way ANOVA demonstrate that PI values are not affected by genotype or treatment variables, while their interaction account for the 17.90% (*p* < 0.024) of the total variance. As far as DI results, genotype significantly account for the 25.92% (0.0016) of the total variance, while InRapa treatment do not affect data. The interaction between genotype and treatment significantly account for the 21.02% (0.0039) of the total variance.

The effects of InRapa on the working and reference memory was further evaluated by the radial arm maze (RAM) test. In 9 days of trial and in the test-day (day 10) we measured three different parameters (i) the time that all mice spend to reach all the 4 beads (Latency, min) (Fig. 2c, d); (ii) the reference memory errors, entry to an empty arm (Fig. 2e, f); (iii) the working memory errors, repeat entries to arms of the maze already visited (Fig. 2g, h). At day 1 all the mice spent an equal time to reach the beads and they showed no significant differences in latency and reference memory errors. At the day 10 Ts65Dn Veh showed poor acquisition ability, measured as increased in latency (50%, *p* = 0.0015) and as well as in working (80%, *p* = 0.042) and reference (40%, *p* = 0.021) memory compared to Eu Veh group. On the other hand, the number of errors (working and reference memory) and the latency was lowest for Eu Veh, Eu InRapa and Ts65Dn InRapa groups and this effect was persistent during testing and was evident especially at day 10. Indeed, if the attention is focused on day 10 (considered as Test Day), Ts65Dn treated with InRapa showed a decreased latency (45%; *p* = 0.07) as show in Fig. 2d and reference memory errors (40%, *p* = 0.013) (Fig. 2h) compared to Ts65Dn Veh. A decreasing trend (not significant) for working memory errors (50%; *p* = 0.12) was evident in Ts65Dn treated with InRapa (Fig. 2f) compared to Ts65Dn Veh. These results showed a partial recovery in cognition for Ts65Dn treated with InRapa. The analysis of RAM results by Two-way ANOVA show that genotype significantly account for the 35.71% (*p* = 0.0004) and the 21.71% (0.0034) of the total variance of latency and reference memory results, respectively, while InRapa treatment significantly account for the 20.91% (*p* = 0.0039) of the total variance of reference memory results. The interaction between genotype and treatment significantly account for the 13.45% (0.0171) of the total variance of reference memory data only.

The results obtained by NOR and RAM tests demonstrate a significant effect of InRapa in Ts65Dn group compared to Veh group, supporting that a targeted rapamycin treatment is able to partially restore memory in Ts65Dn mice.

### InRapa decreases mTOR hyperactivation and induces autophagy

**Fig. 3:**
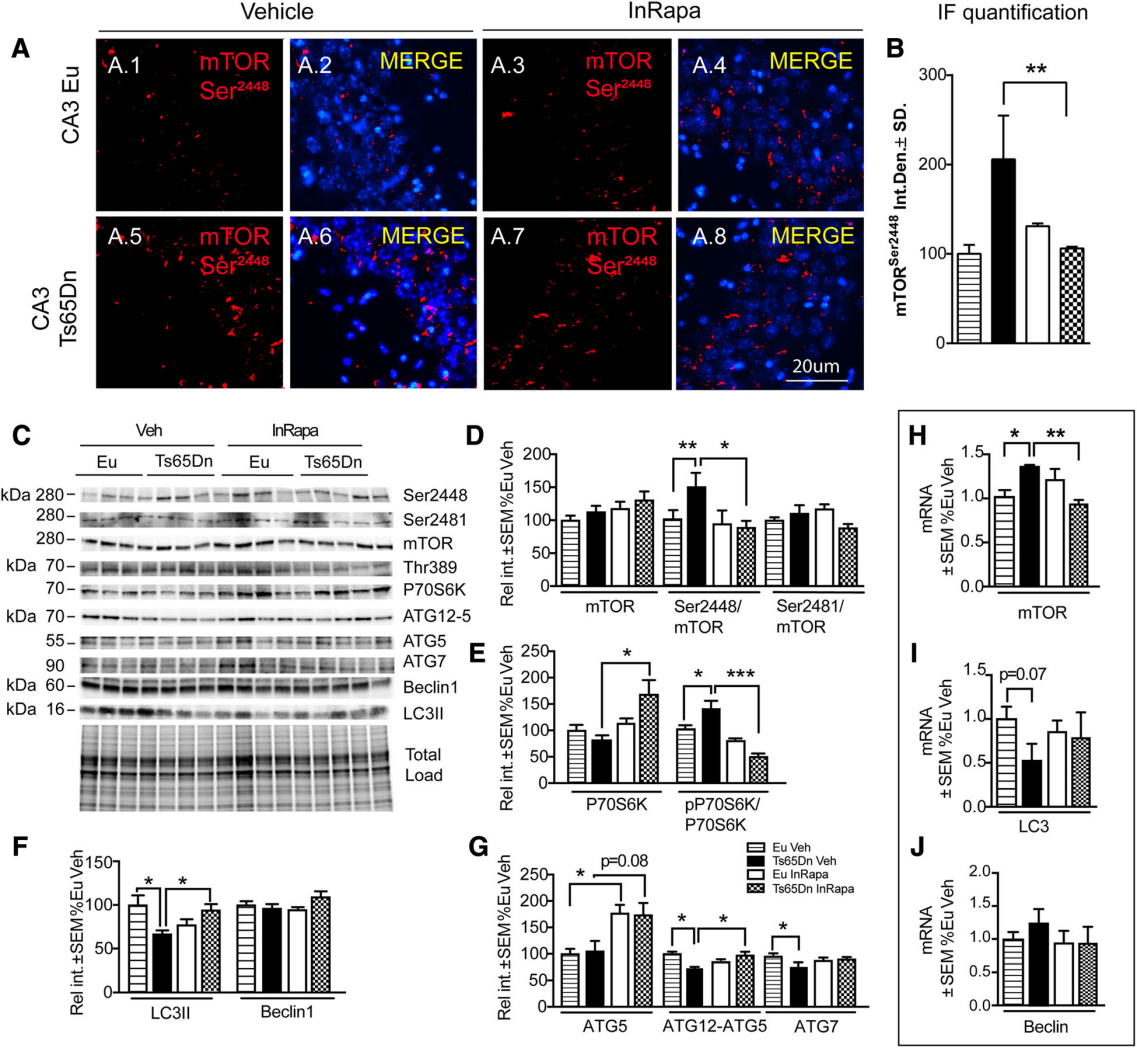
InRapa recovers mTOR hyperactivation and induce autophagy. (**a** 1–8) Representative immunofluorescent images showing p-mTOR(Ser2448) signal in the CA3 region of the hippocampus from euploid mice treated with Veh and InRapa (A1–4), and Ts65Dn mice treated with Veh and InRapa (A5–8). DAPI (blue) was used to identify cell nuclei. Scale bar represent 20 μm. (**b**) Quantification of fluorescence signal. (**c**) Representative WB showing hippocampal p-mTOR (Ser 2448,2481) and mTOR total protein levels, p-P70S6K and P70S6K total protein levels, Atg5, Atg7, Beclin, LC3II protein levels. (**d**) Quantification of panel C showing mTOR protein levels, p-mTOR (Ser2448)/mTOR ratio and p mTOR (Ser2481)/mTOR ratio. (**e**) Quantification of panel C showing P70S6K and p-P70S6K/ P70S6K ratio. (**f**) Quantification of panel C showing LC3II and Beclin protein levels. (**g**) Quantification of panel C showing Atg5, Atg7 protein levels and quantification of the complex Atg5-Atg12. (**h-j**) Quantification of mRNA levels of mTOR (**h**), LC3 (**i**) and Beclin (**j**) analyzed by RT-PCR analysis. Densitometry values shown in the bar graph are the mean of 8 (WB) and 6 (RT-PCR) samples per each group normalized per total load and are given as percentage of Eu Veh, set as 100%. (*p < 0.05, **p < 0.01, ***p < 0.001)

We recently showed a pathological mTOR hyper-activation in Ts65Dn mice at 6 months of age compared with euploid controls [22]. Since long-term mTOR activation leads to neuronal dysfunction and cell death, we hypothesized that inhibition of p-mTOR in Ts65Dn mice would ameliorate the detrimental effects of chronic over-activation. Overall, our data show that intranasal delivery of the mTOR inhibitor rapamycin was able to target and modulate mTOR kinase activity in the hippocampus (Fig. 3 A-D) without affecting body weight as reported in Table 1. In particular, the biochemical analysis performed in the four groups of comparison demonstrate at first that Ts65Dn Veh mice compared with Eu Veh mice show an increase of mTOR phosphorylation at Ser2448 (69%; *p* = 0.036) (Fig. 3d). Similarly, p-mTOR (Ser2448) staining in the neuronal layer of CA3 in Ts65Dn mice Veh is significantly increased compared with Eu mice treated with Veh (101%; *p* = 0.021) (Fig. 3a.1–5, B). The administration of InRapa in Ts65Dn mice is able to partially decrease mTOR phosphorylation at Ser2448 in comparison with Ts65Dn Veh mice (82%; *p* = 0.014) rescuing the activity of mTORC1 to physiological levels as demonstrated by the comparison with Eu mice (Fig. 3d). Such results are confirmed by IF analysis that show a decrease of p-mTOR in Ts65Dn mice after InRapa of about 98% (*p* = 0.002) (Fig. 3a.5–7, B). Accordingly, the analysis of dentate gyrus, by IF, demonstrate a trend of increase of mTOR phosphorylation in Ts65Dn mice Veh compared to euploids, which decrease after the treatment. The two-way ANOVA analysis of mTOR phosphorylation data show that genotype accounts for the 12.46% of the total variance (*p* = 0.022), while InRapa treatment significantly accounts for the 20.38% of the total variance, no significant interaction between the two factor is present. Results on the reduced mTOR activation in Ts65Dn treated with rapamycin are confirmed also by real-time PCR. Indeed, Ts65Dn Veh mice compared with Eu Veh mice show a significant increase of mTOR gene expression (30%; *p* = 0.024); in contrast, rapamycin administration is able to decrease the mTOR gene expression in Ts65Dn by 30% (*p* = 0.019) and this reduction is comparable to Eu groups (Fig. 3h). However, alteration in mTOR gene expression do not yield changes in protein levels between groups. Rapamycin is considered a strong and specific inhibitor of mTORC1 activity, mTORC2 was originally considered insensitive to rapamycin administration. However, prolonged treatment with rapamycin also was shown to be able to inhibit mTORC2 [38]. Our analysis of mTORC2 activity, indexed by phosphorylation of mTOR at Ser2481, show no alteration between Eu and Ts65Dn mice either with or without InRapa administration (10.3% *p* = 0.8 and 21.9% *p* = 0.23 respectively), supporting the low sensitivity of mTORC2 to rapamycin treatment (Fig. 3d).

mTORC1 is directly involved in regulating the activity of two components of the protein synthesis machinery, including the ribosomal S6 kinase 1 (S6 K1) and the eukaryotic translation initiation factor 4E-BP1. Active mTORC1 leads to the phosphorylation of p70S6K at Thr389, which in turn can exert its kinase activity on the S6 ribosomal protein, involved in protein translation [8]. Our data show, that hyperphosphorylated mTORC1 lead to the hyperphosphorylation of p70S6K in Ts65Dn mice compared to Eu animals (57%; *p* = 0.0002), while InRapa, despite an increase of protein levels, is able to reduce p70S6K activation to Eu values, suggesting the full restoration of the mTOR pathway (90%; *p* = 0.0007) (Fig. 3e). The two-way ANOVA analysis shows that genotype do not significantly account for the total variance of p-P70S6K, while InRapa treatment significantly accounts for the 53.30% (*p* < 0.0001). The interaction between genotype and treatment accounts for the 21.14% of the total variance (*p* < 0.001).

In parallel, mTORC1 is a negative regulator of autophagy by directly phosphorylating and suppressing the kinase complex Ulk1/Atg13/FIP200 required to promote autophagosome formation [39]. Autophagy plays a crucial role in the removal of toxic/aggregated proteins, such as Aβ and p-tau aggregates, and damaged organelles. The alteration of autophagy is reported in various neurodegenerative and lysosomal storage disorders and has been extensively demonstrated in DS [39–42]. A common molecular marker to evaluate the rate of autophagosome formation is represented by the ratio of the isoform II to isoform I of LC3, a microtubule associated protein, involved in phagophore elongation and closure [43]. Our results support the idea that, mTORC1 hyper-phosphorylation lead to decreased autophagosome formation as observed by reduced LC3 II (33%; *p* = 0.04) and also, its gene expression (about 50%; *p* = 0.07) in Ts65Dn mice (Veh) compared with Eu animals. InRapa treatment in Ts65Dn was able to rescue the LC3 II protein levels, as demonstrated by its increase about 27% (*p* = 0.012) when compared to Ts65Dn Veh mice, therefore retrieving autophagy function to physiological condition (Eu Veh) (Fig. 3F). The two-way ANOVA analysis shows that genotype significantly accounts for the 17.38% (*p* < 0.034) of the total variance of p-P70S6K, while treatment has not significant effect (*p* = 0.067). The interaction between genotype and treatment accounts for the 35.12% (*p* < 0.001) of the total variance.

In addition, this result is supported by an increasing trend (not significant, *p* = 0.09) for LC3 gene expression in Ts65Dn treated with rapamycin (Fig. 3I). So far, the molecular levels of Beclin1, involved in autophagosome induction, and of Atg7, Atg5 and Atg12/Atg 5 complex, involved in autophagosome elongation, are currently employed as further indices of autophagy induction [43]. We show a significant reduction in Ts65Dn Veh mice compared to Eu Veh for both Atg7 levels (26%; *p* = 0.043) and Atg5/Atg12 complex levels (25%; *p* = 0.0038) (Fig. 3G). InRapa treatment in Ts65Dn was able to recover the alteration of these autophagy-related markers to levels observed in euploid animals, despite Atg7 levels. Indeed, we found a 22% (*p* = 0.0097) increase in Ts65Dn mice InRapa compared with Ts65Dn Veh for Atg12/Atg5 complex levels and a 68% (*p* = 0.08) trend of increase in Ts65Dn mice InRapa compared with Ts65Dn Veh for Atg5 protein levels (Fig. 3g). The analysis of Atg12/Atg5 complex data by two-way ANOVA show, as well as for LC3 II, that genotype significantly accounts for the 10.27% (*p* < 0.0065) of the total variance, while treatment has not significant effects (*p* = 0.086). The interaction between genotype and treatment accounts for the 52.76% of the total variance (*p* < 0.001). No alterations of Beclin1 were observed both in gene expression and protein (Fig. 3J). Therefore, intranasal treatment by inhibiting mTOR phosphorylation allowed the recovery of autophagy-related markers alterations to the levels observed in Eu animals.

### InRapa reduces aberrant APP levels and APP processing

**Fig. 4:**
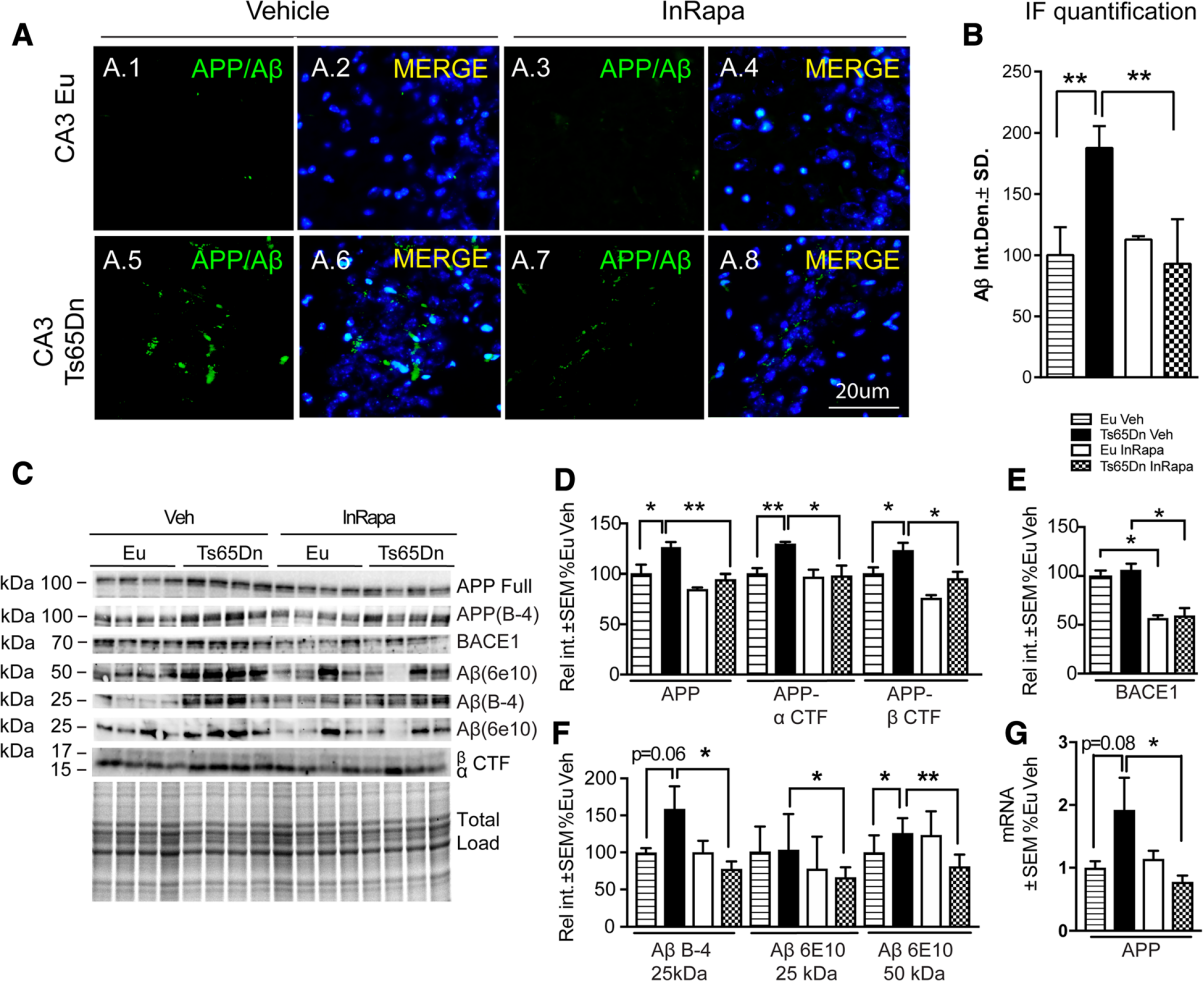
InRapa reduces APP expression levels, APP metabolites and APP processing. (**a** 1–8) Representative immunofluorescent images showing APP/Aβ (B-4) signal in the CA3 region of the hippocampus from euploid mice treated with Veh and InRapa (**a**.1–4), and Ts65Dn mice treated with Veh and InRapa (A.5–8). DAPI (blue) was used to identify cell nuclei. Scale bar represent 20 μm. (**b**) Quantification of fluorescence signal. (**c**) Representative WB showing total hippocampal levels of APP (full and B-4), BACE1, Aβ oligomers (25 and 50 kDa) and β and α CTF. (**d**) Quantification of panel C showing APP, β and α CTF and BACE1 levels. (**e**) Quantification of panel C showing BACE1 levels. (**f**) Quantification of panel C showing Aβ oligomers (25 and 50 kDa) levels. (**g**) Quantification of mRNA levels of APP analyzed by RT-PCR analysis. Densitometry values shown in the bar graph are the mean of 8 (WB) and 6 (RT-PCR) samples per each group, normalized per total load and given as percentage of Eu Veh, set as 100%

The DS population demonstrate that the early accumulation of Aβ peptide plays a key toxic role in the brain resulting in AD-like cognitive decline [1, 2, 5, 44, 45]. Aβ is the product of the proteolytic cleavage of APP, which, among the triplicated genes in DS, is considered the most toxic candidate that contributes to the pathogenesis of AD in DS individuals. The overexpression of APP in DS was shown by previous studies in both humans and mouse samples [4–6, 46]. We analyzed the hippocampus of Ts65Dn and Eu mice after InRapa treatment to investigate changes in APP gene expression, APP protein levels and its metabolites, and Aβ peptide levels. At first, we employed the IF technique with the anti-Aβ (B4) antibody, which recognize both Aβ peptides and APP gene product. This analysis shows an increase of fluorescence in Ts65Dn mice compared to Eu mice (Veh groups) in the CA3 region (90%, *p* = 0.014; Fig. 4a.1–5, B) and in the dentate gyrus (53%, *p* = 0.08). While InRapa treatment decreases the levels of about 103% (*p* = 0.0017; Fig. 4a.5–8, B) in the CA3 region and of about 55% (*p* = 0.027, Additional file 7) in the dentate gyrus. In order to evaluate the contribution of APP or Aβ to IF signal changes, we performed a WB analysis using a different array of antibodies (Fig. 4b). The specific analysis of APP shows, as expected, an increase (26%; *p* = 0.025) in Ts65Dn mice compared to euploids but a restoration of the signal (32% reduction; *p* = 0.0065) in the same animals after InRapa treatment (Fig. 4d). The analysis of Aβ oligomers (at 25 and 50 kD) demonstrated an increase in Ts65Dn mice (about 50% and 30% respectively; *p* = 0.06 and *p* = 0.048), which was significantly reduced by InRapa treatment (70% *p* = 0.035 and 40% *p* = 0.029) (Fig. 4f). These data are particularly intriguing since previous studies showed conflicting results about increased Aβ levels in Ts65dn mice at any age [47–49]. In order to investigate whether the decrease of APP protein levels after InRapa treatment were associated with reduced gene expression, we performed RT-PCR analysis demonstrating the same trend observed by IF and WB analysis: a 90% increase (p = 0.08) in Ts65Dn mice Veh compared to Eu Veh, while a 102% reduction (*p* = 0.031) in Ts65Dn after InRapa administration (Fig. 4g). The two-way ANOVA analysis of Aβ data show that InRapa treatment account for the 14.67% (*p* < 0.0192) of the total variance of Aβ 25 kD oligomers and for the 15.59% (0.0097) of the total variance of Aβ 50 kD oligomers.

Interactions account for the 15.50% (0.0164) and the 26.53% (0.0011) of the total variance of Aβ oligomers at 25 and 50 kD respectively.

The APP processing can follow two different pathways that produce either non-amyloidogenic or amyloidogenic peptides. The non-amyloidogenic pathway is controlled by α-secretases and lead to the formation of s-APPα and α-CTF, while the amyloidogenic pathways lead to the formation of s-APPβ and β-CTF, which can be furtherly cleaved by γ secretase to form Aβ [50]. The analysis of APP processing products demonstrates, in accordance with APP overexpression, the increased formation of α-CTF (29%; *p* = 0.030) and β–CTF peptides (23%; p = 0.048) in Ts65Dn compared to Eu mice treated both with Veh solution. The increased expression of α-CTF and β–CTF, as observed for total APP levels, was recovered significantly by InRapa administration in Ts65Dn mice of about 31% (p = 0.029) and 29% (*p* = 0.018), respectively, when compared to Ts65Dn Veh (Fig. 4D). The two-way ANOVA analysis demonstrate that genotype account significantly for the 17.20% (*p* < 0.0064), the 13.74% (*p* = 0.0103) and the 26.95% (*p* < 0.0001) of the total variance of App, App α-CTF and App β-CTF respectively, while InRapa treatment account for the 29.58% (*p* = 0.0006), the 20.98% (*p* = 0.0021) and the 40.05% (*p* < 0.0001) of the total variance of App, App α-CTF and App β-CTF. The interaction between genotype and treatment is significant only for App α-CTF that account for the 14.42% (*p* = 0.0088) of the total variance.

In addition, the levels of β-secretase (BACE1), the rate-limiting enzyme in β–CTF and Aβ generation, were reduced after rapamycin treatment, in both Ts65Dn and Eu animals, suggesting that its expression is susceptible to rapamycin administration in a strain-independent manner (Fig. 4E). The two-way ANOVA analysis demonstrated indeed that InRapa treatment account for the 49.83% (*p* < 0.0001) of the total variance.

### InRapa modulates tau hyper-phosphorylation and the expression of tau kinases

**Fig. 5:**
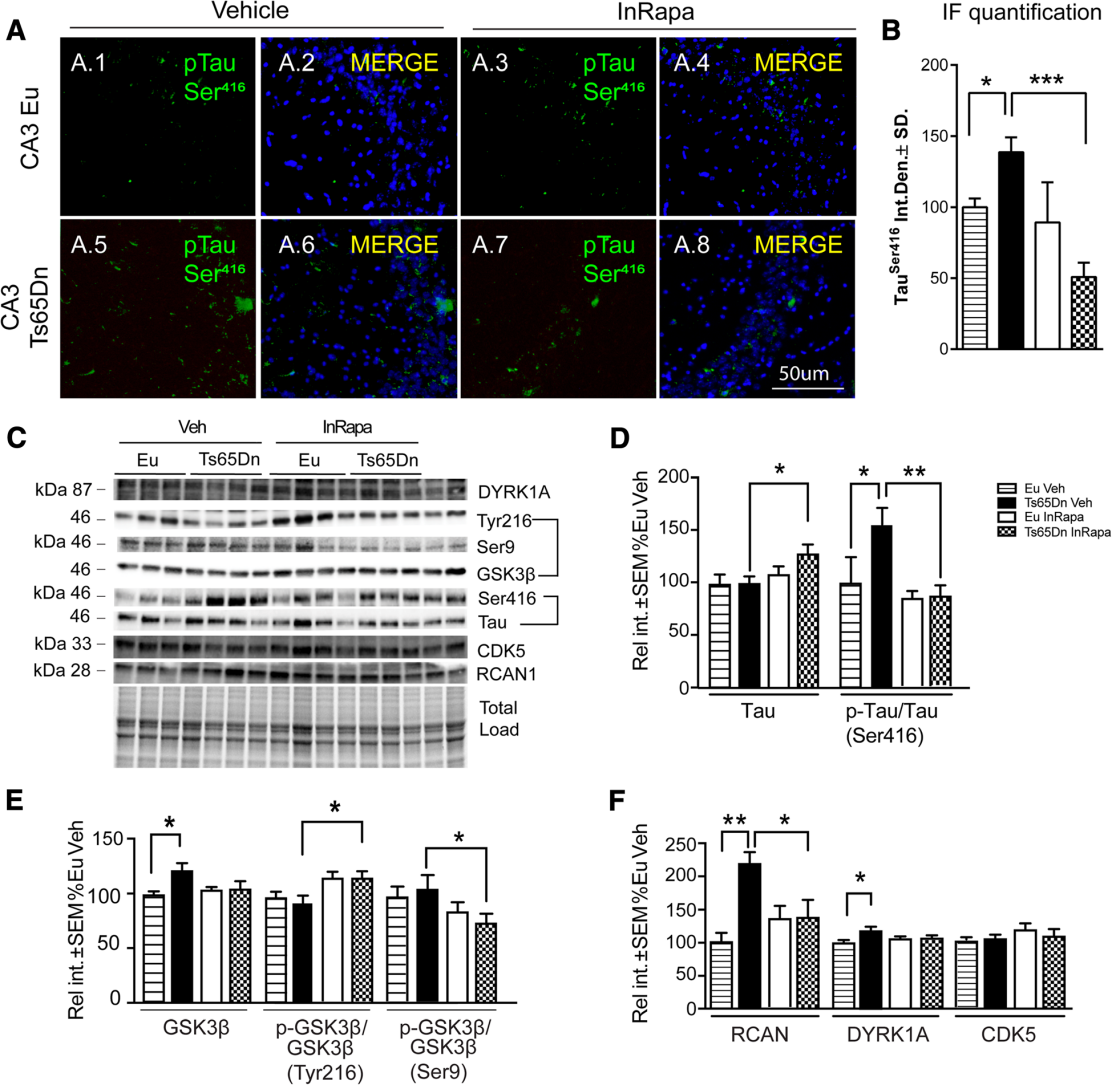
InRapa decreases tau hyper-phosphorylation and expressions of tau kinases. (**a** 1–8) Representative immunofluorescent images showing tau phosphorylation at Ser416 signal in the CA3 region of the hippocampus from euploid mice treated with Veh and InRapa (**a**.1–4), and Ts65Dn mice treated with Veh and InRapa (**a**.5–8). DAPI (blue) was used to identify cell nuclei. Scale bar represent 50 μm. (**b**) Quantification of fluorescence signal (**c**) Representative WB showing hippocampal p-tau (Ser416), total tau levels, GSK3β levels and phosphorylation (Tyr216 and Ser9), DYRK1A, CDK5 and RCAN1 protein levels. (**d**) Quantification of panel C showing levels of tau and p-tau (Ser416)/tau ratio in Eu and Ts65Dn mice treated with InRapa and Veh. (**e**) Quantification of panel C showing GSK3β, p-GSK3β (Ser9)/GSK3β ratio and p-GSK3β (Tyr216)/GSK3β ratio (**f**) Quantification of panel C showing levels of RCAN1, DYRK1A and CDK5 total protein levels. Densitometry values shown in the bar graph are the mean of 8 (WB) samples per each group normalized per total load, and given as percentage of Eu Veh, set as 100%

To further investigate the efficacy of InRapa treatment to reduce AD-related pathological features in DS animals, we examined tau hyper-phosphorylation and the activation of the main kinases involved in its aberrant phosphorylation. The Ts65Dn mice show increased phosphorylation of tau on Ser416 (70%; *p* = 0.027) compared with Eu (Fig. 5d). InRapa treatment on Ts65Dn mice, despite showing a slight increase of levels of tau proteins, demonstrate a robust decrease of tau phosphorylation, in Ser416, when compared to the same mouse strain treated with Veh (80%; *p* = 0.011) (Fig. 5d). Similarly, p-tau (Ser416) staining in the neuronal layer of CA3 (Fig. 5a.1–5, B) and dentate gyrus (Additional file 7) are increased in Ts65Dn Veh mice compared with Eu Veh (45%, *p* = 0.033 and 106%, p = 0.002). InRapa administration in Ts65Dn mice demonstrates the reduction of tau hyper-phosphorylation in Ts65Dn mice both CA3 (90%, *p* = 0.015; Fig. 5a.5–8, B) and dentate gyrus (95%, *p* = 0.004; Additional file 7) regions. The two-way ANOVA analysis of p-tau demonstrated that InRapa treatment, only account for the 16.64% (*p* = 0.0184) of the total variance. Several proteins are involved in tau phosphorylation, such as GSK3β and DYRK1A, that function as direct kinases of tau, or RCAN1 that operate through the inhibition of calcineurin [51–55]. Akt is known to directly regulate GSK3β by phosphorylation of its inhibitory serine residue (Ser9). GSK3β kinase activity on tau phosphorylation, relies on protein levels and on the balance between the phosphorylation of its activatory (Tyr216) and inhibitory (Ser9) residues. GSK3β expression levels were higher in Ts65Dn cases compared to the appropriate Eu treated with Veh (20%; *p* = 0.017). With regard to GSK3β phosphorylation, we show slight but not significant increase of Ser9 and decrease of, while InRapa administration was able to improve GSK3β kinase activity by increasing Tyr216 and decreasing Ser9 phosphorylation (20% *p* = 0.038 and 25% *p* = 0.031) (Fig. 5e). The two-way ANOVA analysis of GSK3β phosphorylation levels show that InRapa treatment account for the 16.33% (p = 0.03) and the 27.60% (0.041) of the total variance of Ser9 and Tyr216 respectively.

As previously noted, tau phosphorylation could be induced directly or indirectly by DYRK1A and RCAN1, which are both encoded on chromosome 21. DYRK1A is expressed in fetal and adult brain and target tau at different serine residues, leading to its aberrant phosphorylation. Tau hyper-phosphorylation occurring in the brain from both Ts65Dn mice and DS subjects correlates with DYRK1A hyperactivation [56]. Our data confirm the over expression of DYRK1A in Ts65Dn mice (19%; *p* = 0.035) due to Chr16 triplication, however this condition was not restored by InRapa administration, (Fig. 5f). DYRK1A data are affected by genotype, which account for the 13.62% of the total variance. Moreover, RCAN1 (regulator of calcineurin 1) is able to control tau dephosphorylation through the regulation of calcineurin. Increased RCAN1 levels result in decreased calcineurin activity and tau hyperphosphorylation. Our data report, as expected, the overexpression of RCAN1 in Ts65Dn Veh compared with Eu Veh (118%; p = 0.004), while InRapa treatment was able to reduce RCAN1 expression levels of Ts65Dn about 79% (% p = 0.03) when compared to Ts65Dn Veh (Fig. 5F). The two-way ANOVA analysis of RCAN1 levels show that genotype treatment account for the 21.52% (*p* = 0.0033) of the total variance. In addition, CDK5 promotes p-tau accumulation in DS [57]. Increased levels of CDK5 was previously reported in the brains of young Ts65Dn [58], however our data show no significant alterations of CDK5 in Ts65Dn mice compared with Eu in both InRapa and Veh groups (Fig. 5f).

### InRapa leads to the recovery of mTOR upstream signalling

mTORC1 is regulated upstream by positive inputs such as growth factors, hormones, chemokines, nutrients (e.g., glucose or amino acids), and cell energy status (ATP/AMP ratio). The regulation of mTORC1 by growth factors mainly involves insulin, which binds to insulin receptor (IR) and triggers the activation of the insulin receptor substrate 1 (IRS1). The phosphorylation of IRS1 on its activatory (Tyr632) or inhibitory (Ser307) residue modulates PI3K activation, which is negatively regulated by PTEN, a phosphatase protein and tensin homolog [8]. PI3K activation lead to increased PIP_3_ levels that recruit Akt, to the membrane, where the latter is activated by phosphorylation of Thr308 and Ser473 residues. In turn, Akt positively regulates mTORC1 activity. Moreover, through a negative-feedback mechanism, mTORC1/p70S6K mediates the inhibitory phosphorylation of IRS1 on a serine residue uncoupling the PI3K/Akt axis from insulin receptor signals [8].

**Fig. 6:**
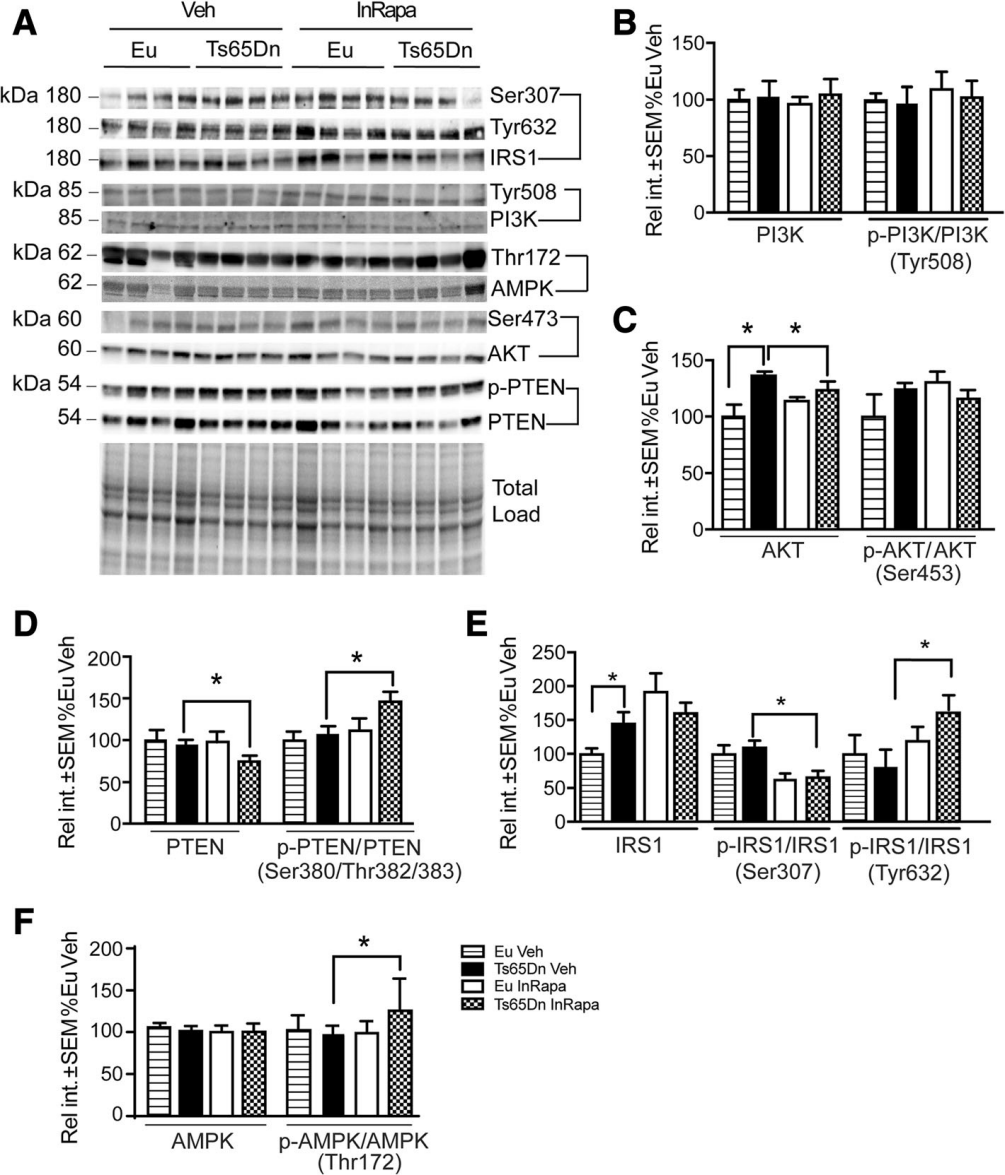
InRapa recovers mTOR upstream signalling. **a** Representative WB showing hippocampal p-IRS (Ser307), p-IRS1 (Tyr632) and total IRS1 levels, p-PI3K(Tyr508) and total PI3K levels, p-AKT (Ser473) and total AKT levels, and p-PTEN (Ser380/Thr382/383) and total PTEN, p-AMPK (Ser172) and total AMPK. **b** Quantification of panel **a** showing hippocampal levels of PI3K and p-PI3K(Tyr508)/PI3K ratio. **c** Quantification of panel **a** showing AKT and p-AKT(Ser473)/AKT ratio. **d** Quantification of panel **a** showing levels of PTEN and p-PTEN (Ser380/Thr382/383)/PTEN ratio. **e** Quantification of panel **a** showing p-IRS1 (Ser307), p-IRS1 (Tyr632) and total IRS1 levels in Eu and Ts65Dn mice treated with InRapa and Veh. **f** Quantification of panel **a** showing AMPK and p-AMPK(Thr172)/AMPK ratio. Densitometry values shown in the bar graph are the mean of 8 samples per each group, normalized per total load and given as percentage of Eu Veh, set as 100%

The analysis of mTOR upstream signalling regulation shows no differences in levels and phosphorylation of PI3K (subunit p85; Tyr508) and increased levels of Akt in Ts65Dn Veh compared to Eu Veh (40%; *p* = 0.022), but no differences in phosphorylation (Ser473) between all groups (Fig. 6b, c). Intriguingly, increased p-PTEN (Ser380/Thr382/383)/PTEN was found to be statistically significant in Ts65Dn InRapa compared with Ts65Dn Veh (37%; *p* = 0.049) suggesting that despite decreased expression, PTEN activation is induced by InRapa, perhaps to better promote a correct regulation of the signal (Fig. 6d). The two-way ANOVA analysis show that genotype and interaction account for the 42.93% (*p* < 0.0001) and for the 14.22% (*p* = 0.005) of the total variance of Akt.

The analysis of the foregoing member of IRS1 pathway demonstrates that, despite increased protein levels of IRS1 in Ts65Dn mice compared to Eu (45%; p = 0.049), a trend of inactivation in Ts65Dn mice, as indexed by decreased phosphorylation of its activation residue (Tyr632) and increased phosphorylation of its inhibitory residue (Ser307), is shown. Rapamycin delivered intranasally is able to reduce the inhibition (exerted by mTORC1 and p70S6K) on IRS1 by increasing its activation residue of phosphorylation (80%; *p* = 0.03) and decreasing its inhibitory phosphorylation (52%; *p* = 0.012) in Ts65Dn InRapa compared to Ts65Dn Veh animals (Fig. 6e). The two-way ANOVA analysis of IRS1 phosphorylation levels show that InRapa treatment account for the 62.94% (*p* < 0.001) and the 13.89% (0.0476) of the total variance of Ser307and Tyr632, respectively.

The AMP-activated protein kinase (AMPK) is a key energy sensor and regulates cellular metabolism to maintain energy homeostasis. AMPK is an upstream signal of mTOR and its activation results in the inhibition of mTOR signalling, thereby suppressing protein synthesis, which is an important pathway by which AMPK conserves cellular energy during low energy states. In turn, prolonged mTOR hyper-phosphorylation on Ser2448 reduces AMPK activation [59]. Ts65Dn mice, in the presence of mTOR hyperphosphorylation, do not show increased pAMPK/AMPK signal, while InRapa treatment was able to reactivate AMPK as indexed by increased activatory phosphorylation on Thr172, of about 30% (p = 0.02) (Fig. 6f).

### InRapa increases the expression levels of STX 1A and PSD95 synaptic proteins

**Fig. 7:**
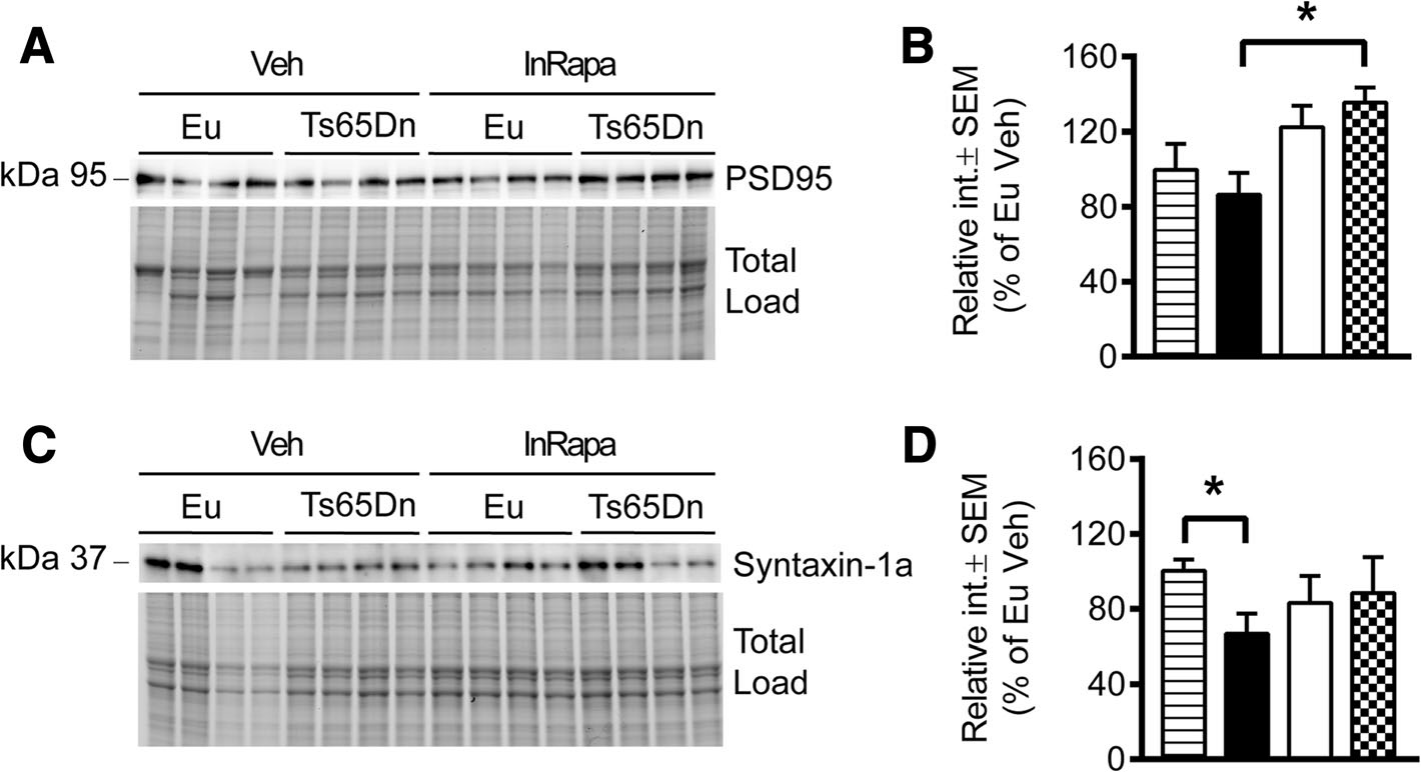
InRapa increases the levels of PSD95 and STX1A synaptic proteins. **a** Representative WB showing hippocampal PSD95 levels. **b** Quantification of panel **a** showing hippocampal the expression levels of PSD95. **c** Representative WB showing hippocampal STX1A levels. **d** Quantification of panel **c** showing levels of STX1A. Densitometry values shown in the bar graph are the mean of 8 samples per each group, normalized per total load and given as percentage of Eu Veh, set as 100%

Prenatal and early post-natal synaptic defects have been largely documented several brain regions including the neocortex, hippocampus and cerebellum of fetuses with Down’s syndrome and of mouse models of the disease [60–64]. Decreased numbers of presynaptic and postsynaptic terminals were previously observed during development in Ts65Dn hippocampus and Ts65Dn dentate gyrus has been shown to have a reduced number of neurons and deficient LTP [60, 65, 66]. To determine whether InRapa treatment result in the rescue of synapse failure, we examined the expression levels of syntaxin1A (STX1A) and PSD 95 in euploid and Ts65Dn mice. STX1A is neuronal plasma membrane protein that belongs to the soluble N-ethylmaleimide-sensitive factor attachment protein receptor (SNARE) family and is involved in vesicle trafficking, docking and/or fusion, playing a key role in neurotransmitter release. PSD 95, a membrane-associated guanylate kinase, is the major scaffolding protein in the excitatory postsynaptic density (PSD) and a potent regulator of synaptic strength. Thought the phosphorylation of 4EBP1 and p70S6K mTOR is able to regulates protein synthesis, influencing the expression of synaptic proteins. Our results show for STX1A expression a significant decrease in Ts65Dn Veh compared to euploid animals (35%, *p* = 0.0137), which is partially rescued (20%, ns) after rapamycin administration (Fig 7a-b). As far as PSD 95 we demonstrate a trend of decrease for its expression levels in Ts65Dn Veh mice compared to euploid Veh (18%, ns), while rapamycin treatment induces the overexpression of PSD 95 in both Ts65Dn (50%, *p* = 0.045) and Euploids (22%, ns) (Fig. 7c-d). Our results are in line with previous studies showing reduced levels of synaptic protein in Ts65Dn mice and their induction after disease-modifying treatment leading to improved cognition [67–69]. Intriguingly, the two-way anova analyses of PSD95 show indeed that the treatment account for the 37.7% (*p* = 0.0077) of the total variance.

### InRapa modulates protein oxidative damage and Lys63 poly-ubiquitinylation

**Fig. 8:**
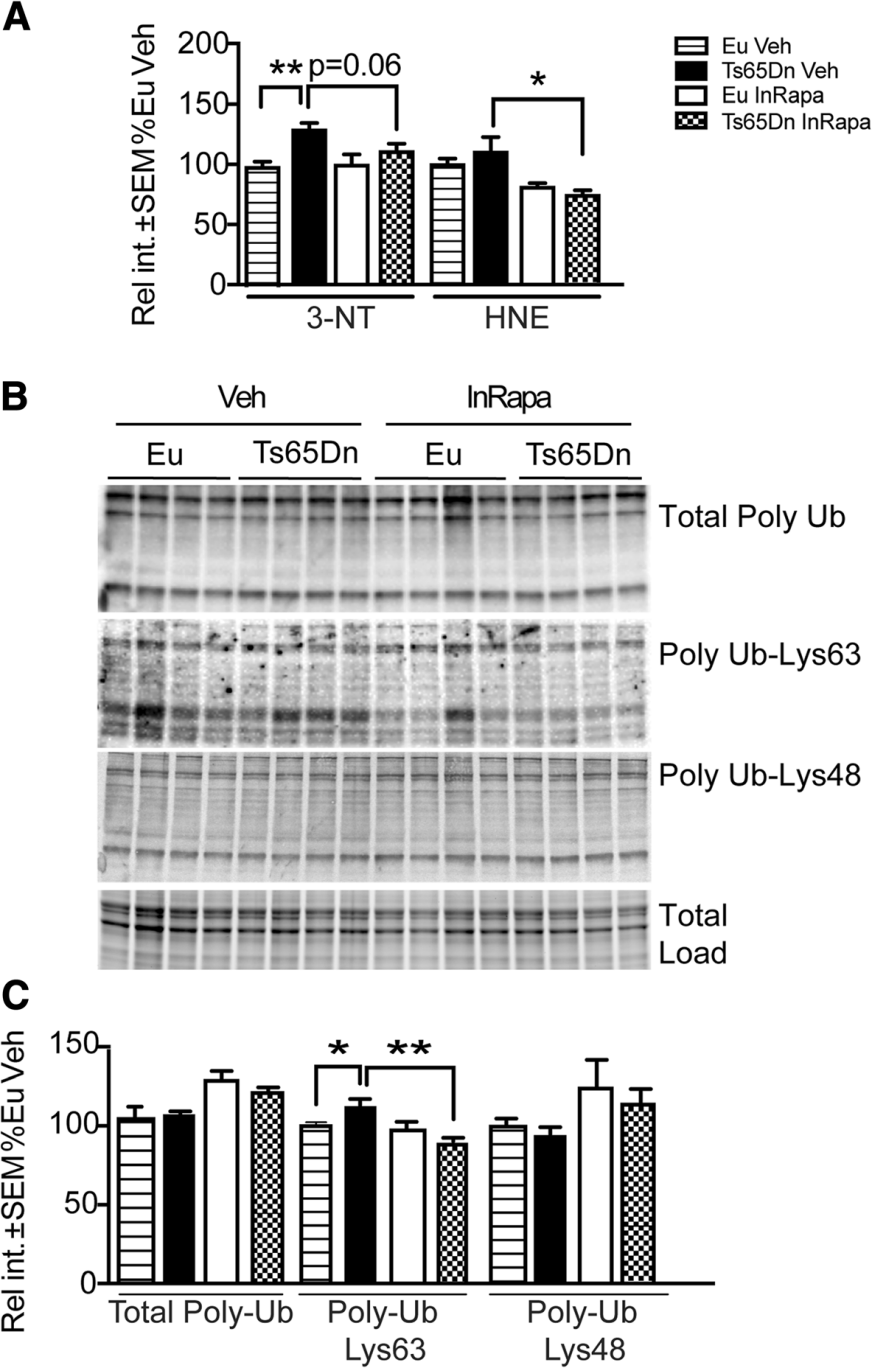
InRapa reduces protein oxidative damage and K63 poly-ubiquitinylation. In panel **a** are shown the total hippocampal protein-bound 3-NT and HNE of Ts65Dn mice and Eu treated with Veh and InRapa analyzed by slot blot assay. In panel **b** are shown the hippocampal total poly-ubiquitin, poly-ubiquitin Lys63 and poly-ubiquitin Lys48 levels analyzed by Western blot. In panel **c** the quantification of panel **b** is reported. Densitometry values of each lane is the result of the sum of all the bands, analyzed by Image lab software as previously reported [10]. Graph values are the mean of 8 samples per each group, normalized per total load and given as percentage of Eu Veh, set as 100%

Previous studies by our group and others demonstrated that in DS brain alteration of autophagy is associated with increased oxidative stress (OS), which plays an important role in DS neuropathology [7, 9, 13, 22, 70]. However, the link between OS and autophagy is intricate, and increasing evidence suggests that the mTOR/autophagy axis plays a dual role in the cellular response to OS [71, 72] . We evaluated the levels of two protein oxidation markers, 4-hydroxy-2-nonenal protein adducts (HNE) and protein-bound 3-nitrotyrosine (3-NT) in the hippocampus of Ts65Dn and Eu. We found a significant elevation of total 3-NT levels in Ts65Dn mice compared with Eu treated with Veh (28%; *p* = 0.021), and such increase was reduced with InRapa treatment (23%; *p* = 0.06) (Fig. 8a). Further, a trend of increased HNE adducts to proteins was observe between Eu Veh and Ts65Dn Veh, and treatment with InRapa was able to significantly reduce such increase (43%; *p* = 0.015) (Fig. 8a). The two-way ANOVA analysis of 3NT levels show that genotype significantly account for the 31.05% (*p* = 0.0007) of the total variance, while if we consider protein-bound HNE levels InRapa treatment and interaction account for the 47.01 (*p* < 0.0001) and for the 7.19% (*p* = 0.043) of the total variance, respectively.

We also investigated the status of the ubiquitin-dependent proteolysis pathway through analysis of total protein poly-ubiquitinylation and the levels of chain linkage Lys48, considered to be a key signal for proteasome degradation, and chain linkage Lys63, known to have a role in protein degradation through the autophagy-lysosomal system [73]. However, we showed that Lys63 protein poly-ubiquitinylation might have a primary role in protein signalling in addition to protein degradation [10]. With regards to total protein poly-ubiquitinylation, no significant alteration was observed between DS and Eu animals with and without rapamycin treatment (Fig. 8c). However, if we discriminate the lysine residue of poly-ubiquitinylation we observed increased levels of Lys63 poly-ubiquitinylated proteins in Ts65Dn Veh mice compared to Eu Veh (13%; *p* = 0.024), which are reduced in Ts65Dn InRapa compared to Veh (24%; *p* = 0.0012) (Fig. 8c). No significant changes were detected, among groups, for Lys48 poly-ubiquitinylated proteins. The two-way ANOVA analysis of Lys63 poly-Ub levels demonstrate that InRapa treatment and interaction account for the 32.71 (*p* = 0.0001) and for the 20.84% (*p* = 0.0013) of the total variance, respectively.

## Discussion

In the last decades, a significant increase of life expectancy has been observed in DS individuals due to improvement in health care. However, improved lifespan in DS is associated with an increased incidence of developing AD-like dementia [2, 74]. The triplication of APP represents a strong evidence on the influence of HSA21 trisomy in the progression to AD-like cognitive decline in DS population. In addition, the triplication of tau kinases, such as DYRK1A and RCAN1, which act in parallel with aberrant mTOR pathway activation, contributes to increased tau phosphorylation and NFT formation [72, 75, 76]. The exact mechanisms by which triplication of genes on HSA21 lead to the early onset of AD in DS population remain still to be fully elucidated.

Previous studies by our group and others demonstrated the hyper-activation of mTOR pathway in human brain from DS population and in Ts65Dn mouse model of the disease [13, 21, 22, 56, 77]. mTOR hyper-activation was found to be strongly associated with reduced autophagosome formation (most likely leading to impaired autophagy induction), increased Aβ deposition and increased tau hyper-phosphorylation. Data collected by our studies support the key role of aberrant mTOR signalling in mediating the early progression of AD in DS population. Within this frame, the rescue of mTOR signalling by the administration of rapamycin, which has been previously tested in AD mouse models demonstrating favorable outcomes [19, 27, 29–31, 78–81], represents a potentially valuable therapeutic strategy. Indeed, evidence obtained by the Oddo and Galvan laboratories [19, 31] corroborated the positive effects of mTOR inhibition on hippocampal memory rescue in AD mice. In particular, the authors reported that chronic oral rapamycin treatment was able to prevent cognitive loss in two different transgenic mouse models of AD, 3xTg-AD and J20, if given before robust plaque and tangle deposition. Memory improvement was associated with reduced Aβ levels and tau-aggregation, as well as microglial activation [19, 31]. Based on these evidences, the initiation of an early treatment schedule is necessary to achieve brain protection in DS individuals, known to be at high risk of developing AD-like dementia. Moreover, the use of the intranasal route for the delivery of rapamycin to the brain holds a great potential as a non-invasive practical approach that circumvents systemic alterations and permits to maximal drug concentrations in the CNS, thereby avoiding the rapamycin-related immunosuppressant effects in the periphery [82–85]. This issue is particularly critical in DS pathological phenotypes, since trisomic individuals show depletion of immune system and lymphopenia [86–89]. In this scenario, by UPLC-MS analysis we were able to demonstrate that intranasal administration of rapamycin concentrated the drug in the CNS, where it exerted its inhibitory effects by reducing hippocampal mTORC1 hyper-phosphorylation of about 50%, while, as expected, no effects were observed in peripheral organs analyzed. Of note, the rapamycin dosage delivered to the hippocampus of Ts65Dn was selected to not abolish mTOR activation but to rescue the signal at physiological levels, thus abrogating the pathological increase of mTOR and p70S6K phosphorylation along with the reduction of autophagy.

The restoration of mTORC1 activity after treatment demonstrated a significant effect on cognitive performance in Ts65Dn mice as indexed by RAM and NOR tests. Indeed, the RAM test revealed that mice were able to improve reference and working memory after rapamycin treatment. As well, the NOR test showed the improvement of mice preference index supporting the recovery of novelty-discriminating ability after rapamycin administration. Further, we suggest that the improved cognition, exerted by InRapa, is associated with the rescue of synaptic abnormalities previously observed in DS [60, 69]. Therefore, as previously proven in AD, our data support the capability of rapamycin, if delivered chronically and intranasally before consistent brain damage, to improve memory and reduce cognitive decline in DS mice [31]. In order to understand the mechanisms leading to improved cognition after InRapa treatment we investigated the status of downstream targets of mTOR and the pathological features of AD-like neurodegenerative process.

Human and mouse studies suggest that APP is dosage sensitive as a function of aging and of brain regionalism [4, 6, 46, 50]. In contrast, Aβ accumulation becomes significant in humans only in the second/third decade of life, with some exceptions in a few childhood post- mortem observations [2, 75, 76]. Moreover, published data on Aβ peptides levels in the brain of Ts65Dn mice are conflicting. This discrepancy might depend on the diversity of techniques, brain area analyzed and age of the samples. We observed an increase of Aβ oligomers at 25 and 50 kD only, while previous studies focused on the identification of plaques, which are formed only at a very late stage of the disease. Within this scenario, our data suggest a primary role for APP and its processing in the neurodegenerative process occurring in DS [33, 47, 49, 50, 90]. Indeed, in Ts65Dn mouse, which develops AD-like neuronal endosomal pathology, the increase of APP-αCTF and APP-βCTF between 6 and 12 months of age is likely to underlie the failure of NGF-mediated trophic support [6], and contribute to cognitive failure. Our analysis shows increased levels of APP in Ts65Dn mice at 9 months of age both in total hippocampus as well as in the CA3 region, together with the increased APP metabolites APP-αCTF and APP-βCTF. InRapa administration is able to significantly reduce APP levels in the hippocampus of Ts65Dn mice and to decrease APP metabolites, suggesting the re-establishment of proper APP processing. Surprisingly, we also observed increased Aβ oligomers in Ts65Dn mice and the reduction of such increase due to InRapa treatment. Two main mechanisms can be directly involved in the reduction of APP, APP metabolites and Aβ in Ts65Dn mice after rapamycin treatment: i) the reduction of APP gene expression; ii) the rescue of protein synthesis/degradation pathways; ii) the restoration of key signalling pathways, including BACE1, PI3K/Akt, GSK-3β, AMPK and IRS1, that regulate APP processing products formation/clearance [8, 12, 14, 39, 72, 91] . Our data show, as expected, the increase of APP mRNA in Ts65Dn mice as a result of trisomy that demonstrate a significant decrease after InRapa treatment. In general, mTORC1 inhibition by rapamycin results in a reduction in global mRNA, indeed mTOR is able to bind a number of transcription factors (e.g. STAT3; PGC1α) that can regulate the expression of a broad range of target genes, comprising mTOR itself, whose aberrant modulation is known to be involved in neurodegeneration [92]. In agreement, our data supports a role for rapamycin in the down-regulation of APP transcription process. The transcription factor ETS2, encoded on Chr21, was demonstrated to transactivate the APP promoter, leading to its overexpression [93]. Previous studies revealed that the expression levels of ETS2 can be modulated by the mTOR pathway; therefore, rapamycin-induced mTOR inhibition, through the reduction of ETS2 levels, might reduce APP overexpression levels [94]. Besides, it is indeed equally important to highlight the significant increase of autophagosome formation observed in Ts65Dn mice after InRapa treatment. Our data demonstrate that mTOR inhibition lead to increased LC3II and Atg 12/5 levels supporting a crucial role for rapamycin in restoring the aberrant control of mTOR on autophagy. The observed induction of autophagosome formation in rapamycin treated DS mice is associated with the reduction of toxic aggregates burden and misfolded/oxidized proteins accumulation. Therefore, our results are in agreement with previous studies demonstrating that rapamycin-dependent stimulation of autophagy is likely one of the principal mechanisms by which the reduction of toxic protein aggregates, comprised of Aβ, aberrantly expressed APP and APP metabolites, is achieved in the brains of Tg-AD mice [18, 19, 23, 26, 31, 40, 79, 95]. Furthermore, the analysis of molecular pathways involved in APP processing demonstrated that rapamycin lead to the reduction of BACE1 levels and to the recovery of IRS1 signaling. Conversely, we obtained conflicting results concerning PI3K/Akt and GSK3β that suggest their modest involvement in APP metabolite reduction. The increased BACE1 activation is required for the cleavage of APP and the production of the neurotoxic Aβ peptide during neurodegeneration, as demonstrated in AD mice and in our DS model [4, 5]. On one side we could suppose that the reduction of BACE1 levels, observed after InRapa treatment, could be most likely related with the reduction of the APP substrate, accomplished by re-balanced synthesis/degradation. However, because such reduction is observed in both Ts65Dn and Eu mice, it is tempting to presume a close interaction between rapamycin/ mTOR and BACE1. The direct interaction of mTOR with IRS1 have been previously demonstrated and was shown to be deeply involved in the development of AD [13, 20, 21, 96–98]. Indeed, the failure of energy metabolism associated with the increase of brain insulin resistance are well-recognized contributors to AD neurodegeneration [96, 97, 99]. Our previous studies demonstrated that mTOR hyperphosphorylation and the subsequent overactivation of p70S6K kinase activity target IRS1 by increasing the phosphorylation on its inhibitory serine residues, which lead to the inactivation of the protein. Such effects contribute to the uncoupling of IRS1 from PI3K/Akt signalling and to the development of brain insulin resistance [13]. The rescue of mTOR signalling in Ts65Dn mice after InRapa leads to reduced IRS1 inhibitory phosphorylation sites; therefore, a proper insulin signalling is reinstated that contributes to decreased metabolic failure and conceivably reduced APP processing products in DS [84, 97, 100, 101].

As noted above, AMPK signalling is a major inducer of autophagy associated with the reduction of energy metabolism [59]. The loss of sensitivity of AMPK activation to cellular stress impairs metabolic regulation, increases oxidative stress and reduces autophagic clearance. Recent studies confirmed that the responsiveness of AMPK to different insults is clearly suppressed in aged tissues during mTOR overactivation. In line with this proposed scenario, AMPK signal is dampened in Ts65Dn mice. Intriguingly, the inhibition of mTOR, by InRapa treatment, lead to increased phosphorylation of AMPK on its activatory residue rescuing signalling induction. Noteworthy, previous reports demonstrated that AMPK activation is able to induce autophagy by also phosphorylating Ulk1, beyond inhibiting mTOR signalling [102].

The sequence of pathological mechanisms of DS neurodegeneration in Ts65Dn mice include aberrant tau phosphorylation, associated with the increased activation state of different tau kinases. Among these, DYRK1A and RCAN1 encoded on HSA21, and GSK3β and CDK5 seem to have a prominent role in tau hyper-phosphorylation occurring in the brain of AD and DS subjects [72].

Substantial evidence supports the critical role of mTOR in tau-related pathological progress in DS. A number of studies sustain that the activation of mTOR signalling promotes tau hyper-phosphorylation, while its inhibition prevents this phenomenon [27–29, 32, 95]. In particular, the mechanisms by which altered mTOR signalling lead to tau hyper-phosphorylation include the aberrant regulation of tau kinases and the reduction of autophagy. Ts65Dn mice after InRapa treatment demonstrate a robust and significant reduction of tau phosphorylation in the hippocampus, both total and CA3 region-specific. The reduction is associated with significantly decreased expression of RCAN1 and with a trend of decreased DYRK1A levels. No alteration is shown for CDK5 in Ts65Dn prior or after the treatment, while an opposite trend is reported for GSK3β, as previously reported also in human studies [13]. Despite the reduction of tau kinases, lowered levels of hyperphosphorylated tau cannot be observed after InRapa administration without the increase of autophagosome formation, which play a key role in the clearence of intracellular toxic tau aggregates. These results suggest that rapamycin is able to reduce the degree of tau phosphorylation by modulating the expression of certain tau kinases, and to improve hyperphosphorylated tau degradation through the induction of autophagy [103–105].

A further intriguing outcome observed after InRapa treatment is represented by the reduction of protein oxidative damage in Ts65Dn mice. Increased oxidative stress is a characteristic feature of DS neuropathology in humans and mice [3, 74, 106]. Data collected in DS human brain indicated that oxidative damage targeted specific components of the proteostasis network, resulting in dysfunctional activation of autophagy and the ubiquitin proteasome system [7, 9, 70]. Previous studies by our laboratory demonstrated that the chronic increase of OS in Ts65Dn mice with aging, in parallel with reduced autophagy, leads to the accumulation of total protein-bound HNE and protein nitration levels [22]. Therefore, a vicious cycle that involves the prolonged failure of protein degradation systems and the chronic build-up of oxidized protein may exist in DS. The rescue of mTOR activity, by InRapa treatment, likely induces the autophagy-driven degradation of oxidized proteins as demonstrated through the decrease of total protein-bound HNE levels and total protein nitration. In addition, studies on DS human brain reported that the accumulation of oxidative damage is coupled with increased levels of poly-ubiquitinylated proteins, prior to and after the development of AD [10]. Despite, Ts65Dn did not recapitulate the same profile of protein poly-ubiquitinylation observed in humans an increase in poly-Lys63 ubiquitinylation levels were observed. These data suggest that impairment of the proteasome system in Ts65Dn mice is less pronounced compared with humans, supporting the concept that the failure of protein degradation is related to the impairment of the autophagy pathway rather than to the impairment of the UPS. Noteworthy, InRapa treatment was able to reduce the poly-ubiquitinylation of Lys63 residues supporting the restoration of autophagy and implying a certain degree the crosstalk between mTOR and UPS.

## Conclusions

Overall, we demonstrated that rapamycin, administered for 3 months by intranasal route, led to improved cognition in DS mice with no effects at peripheral organs. The favorable outcomes of rapamycin treatment seem to rely on its ability to rescue molecular pathways associated with aberrant mTOR phosphorylation, whose alteration accelerate the age-related neurodegenerative process and increase the risk of AD development in DS. Therefore, InRapa treatment represents an attractive therapeutic strategy to reduce the early development of neuropathology in DS population and delay the onset of AD. At final, this therapeutic strategy may be also translated to different neuronal disorders that share, as a common pathological mechanism, the alteration of mTOR/autophagy axis.

## Additional files

Additional file 1: List of antibodies used for WB and IF analysis. For each antibody employed in the study is reported the brand, the catalogue number and the dilution employed in the study. (PPTX 146 kb)

Additional file 2: Bar graph reporting mice distance traveled during RAM test. The distance travelled was measured for 10 min at the end of the test days in all experimental groups, no differences were found between the groups. (PPTX 2553 kb)

Additional file 3: Pilot studies to assess InRapa therapeutic dose. Mice were treated by InRapa daily for 1 week after which brain regions were collected and analyzed. Phosphorylation levels of mTOR in both hippocampus and cortex are reported for each of the dose tested, 0.01 μg/μl (0.1 μg/mouse), 0.05 μg/μl (0.5 μg/mouse), 0.1 μg/μl (1 μg/mouse) and 0.2 μg/μl (2 μg/mouse). Each value is the mean of 6 replicate ± SEM. Our data demonstrate that the InRapa dosage of 0.1 μg/μl (1 μg/mouse) is able to inhibit mTOR phosphorylation when compared to vehicle. (PPTX 1203 kb)

Additional file 4: Table reporting 2-way ANOVA data analysis. For 2-way ANOVA analysis only proteins showing changes before and after the InRapa treatment have been taken under consideration. (PPTX 844 kb)

Additional file 5: Rapamycin distribution by UPLC-MS. Chromatograms of rapamycin in plasma (A) and brain (B) from animals treated by single I.P. injection of 50 μg/mouse (2,5 mg/kg/mouse) 4 h before sacrifice. Chromatograms of rapamycin in plasma (C) and brain (D) from animals treated by single InRapa administration of 1 μg/mouse (0.05 mg/Kg/mouse) 4 h before sacrifice. (PPTX 50 kb)

Additional file 6: Western blot analysis of mTOR and p70S6K phosphorylation in liver and heart tissue after InRapa treatment. Graph bars are reported as percentage in respect to euploid vehicle group, which is set as 100%. Data Show no significant alteration in Ts65Dn undergoing rapamycin (black bar) or vehicle (checquered bars) after intranasal delivery supporting no effects of InRapa treatment at peripheral level. (PPTX 72 kb)

Additional file 7: Immunofluorescence staining of Dentate gyrus in Eu and Ts65Dn mice. Representative immunofluorescent images showing (A) p-mTOR at serine 2448, (B) at Ser416 and (C) APP/Ab levels in the dentate gyrus region of the hippocampus from euploid mice treated with Veh and InRapa (A.1–4), and Ts65Dn mice treated with Veh and InRapa (A.5–8). DAPI (blue) was used to identify cell nuclei. Scale bar represent 20 μm. On the right of each panel a graph of the quantification of fluorescence signal is reported. (PPTX 16410 kb)

## Abbreviations

3-NT: 3-Nitrotyrosine
AD: Alzheimer’s Disease
AMPK: AMP-activated protein kinase
APP: Amyloid Precursor Protein
Atg: Autophagy related proteins
Aβ: Amyloid-β
BACE1: Beta-secretase 1
CDK5: cyclin dependent kinase 5
CNS: Central Nervous System
Ct: threshold cycle
DI: Discrimination Index
DS: Down Syndrome
DYRK1A: Dual specificity tyrosine-phosphorylation-regulated kinase 1A
Eu: Euploid
GAPDH: Glyceraldehyde-3-Phosphate Dehydrogenase
GSK3β: glycogen synthase kinase 3 isoforms β
HNE: 4-Hydroxynonenal
Hsa21: human chromosome 21
InRapa: Intranasal Rapamycin
IRS-1: Insulin Receptor Substrate-1
LC3: Microtubule-associated protein 1A/1B-light chain 3
mTOR: mammalian Target Of Rapamycin
NFT: Neurofibrillary Tangles
NOR: Novel Object Recognition
OS: Oxidative Stress
P70S6K: ribosomal p70S6 kinase
Pdebrd1: Recessive Retinal Degeneration 1 Mutation
PI: preference Index
PI3K: Phosphoinositide-3-Kinase
PQC: Protein Quality Control
PTEN: phosphatase and tensin homolog
RAM: Radial Arm Maze
Rapa: Rapamycin
RCAN1: Regulator of Calcineurin 1
RME: reference memory errors
RNS: Reactive Nitrogen Species
ROS: Reactive Oxygen Species
RT-PCR: Real time Polymerase Chain Reaction
Tg: Transgenic
UPLC: Ultra-performance/pressure Liquid Chromatography analysis
UPS: Ubiquitin Proteasome System
Veh: Vehicle
WME: working memory errors
