# Supplementary material for: Intranasal rapamycin ameliorates Alzheimer-like cognitive decline in a mouse model of Down syndrome
Source: Transl Neurodegener. 2018 Nov 6;7:28. doi: 10.1186/s40035-018-0133-9 (PMC6218962; doi:10.1186/s40035-018-0133-9)
Supplement: Supplementary file 1 — List of antibodies used for WB and IF analysis. For each antibody employed in the study is reported the brand, the catalogue number and the dilution employed in the study. (PPTX 146 kb) [file 40035_2018_133_MOESM1_ESM.pptx]

## Slide 1
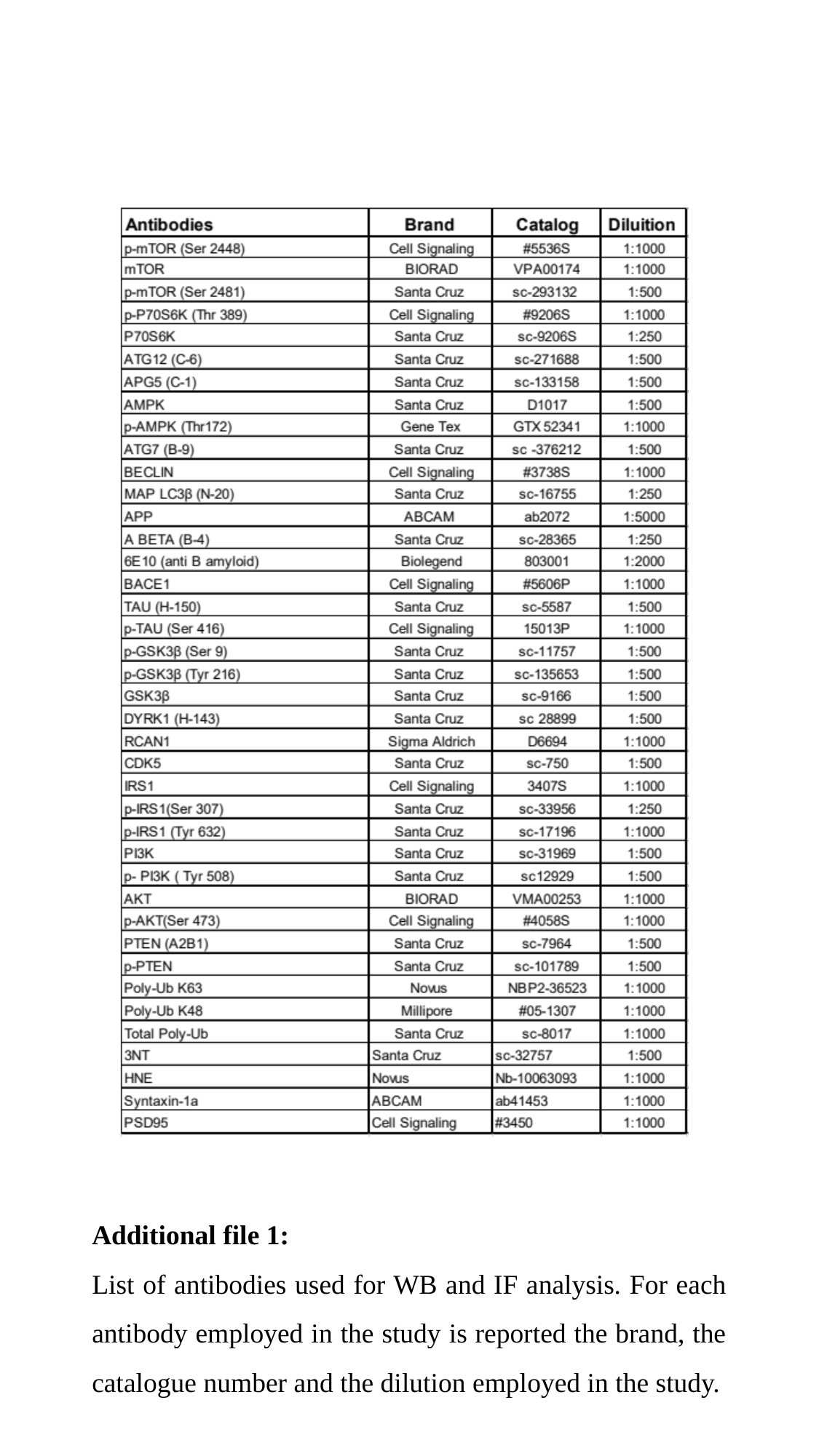

Additional file 1:
List of antibodies used for WB and IF analysis. For each antibody employed in the study is reported the brand, the catalogue number and the dilution employed in the study.
