# Supplementary material for: Intranasal rapamycin ameliorates Alzheimer-like cognitive decline in a mouse model of Down syndrome
Source: Transl Neurodegener. 2018 Nov 6;7:28. doi: 10.1186/s40035-018-0133-9 (PMC6218962; doi:10.1186/s40035-018-0133-9)
Supplement: Supplementary file 2 — Bar graph reporting mice distance traveled during RAM test. The distance travelled was measured for 10 min at the end of the test days in all experimental groups, no differences were found between the groups. (PPTX 2553 kb) [file 40035_2018_133_MOESM2_ESM.pptx]

## Slide 1
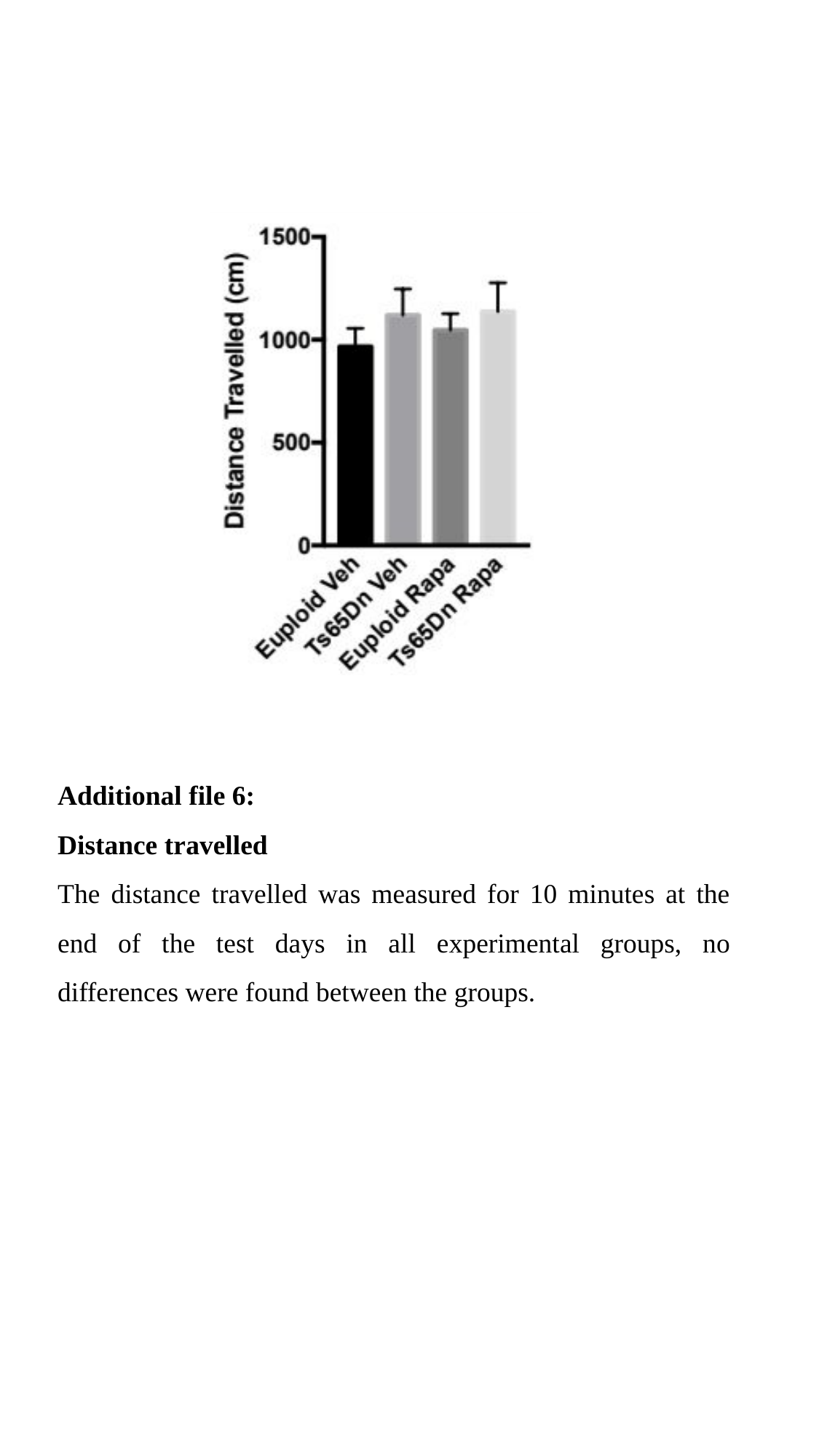

Additional file 6:
Distance travelled
The distance travelled was measured for 10 minutes at the end of the test days in all experimental groups, no differences were found between the groups.
