# Supplementary material for: Intranasal rapamycin ameliorates Alzheimer-like cognitive decline in a mouse model of Down syndrome
Source: Transl Neurodegener. 2018 Nov 6;7:28. doi: 10.1186/s40035-018-0133-9 (PMC6218962; doi:10.1186/s40035-018-0133-9)
Supplement: Supplementary file 3 — Pilot studies to assess InRapa therapeutic dose. Mice were treated by InRapa daily for 1 week after which brain regions were collected and analyzed. Phosphorylation levels of mTOR in both hippocampus and cortex are reported for each of the dose tested, 0.01 μg/μl (0.1 μg/mouse), 0.05 μg/μl (0.5 μg/mouse), 0.1 μg/μl (1 μg/mouse) and 0.2 μg/μl (2 μg/mouse). Each value is the mean of 6 replicate ± SEM. Our data demonstrate that the InRapa dosage of 0.1 μg/μl (1 μg/mouse) is able to inhibit mTOR phosphorylation when compared to vehicle. (PPTX 1203 kb) [file 40035_2018_133_MOESM3_ESM.pptx]

## Slide 1
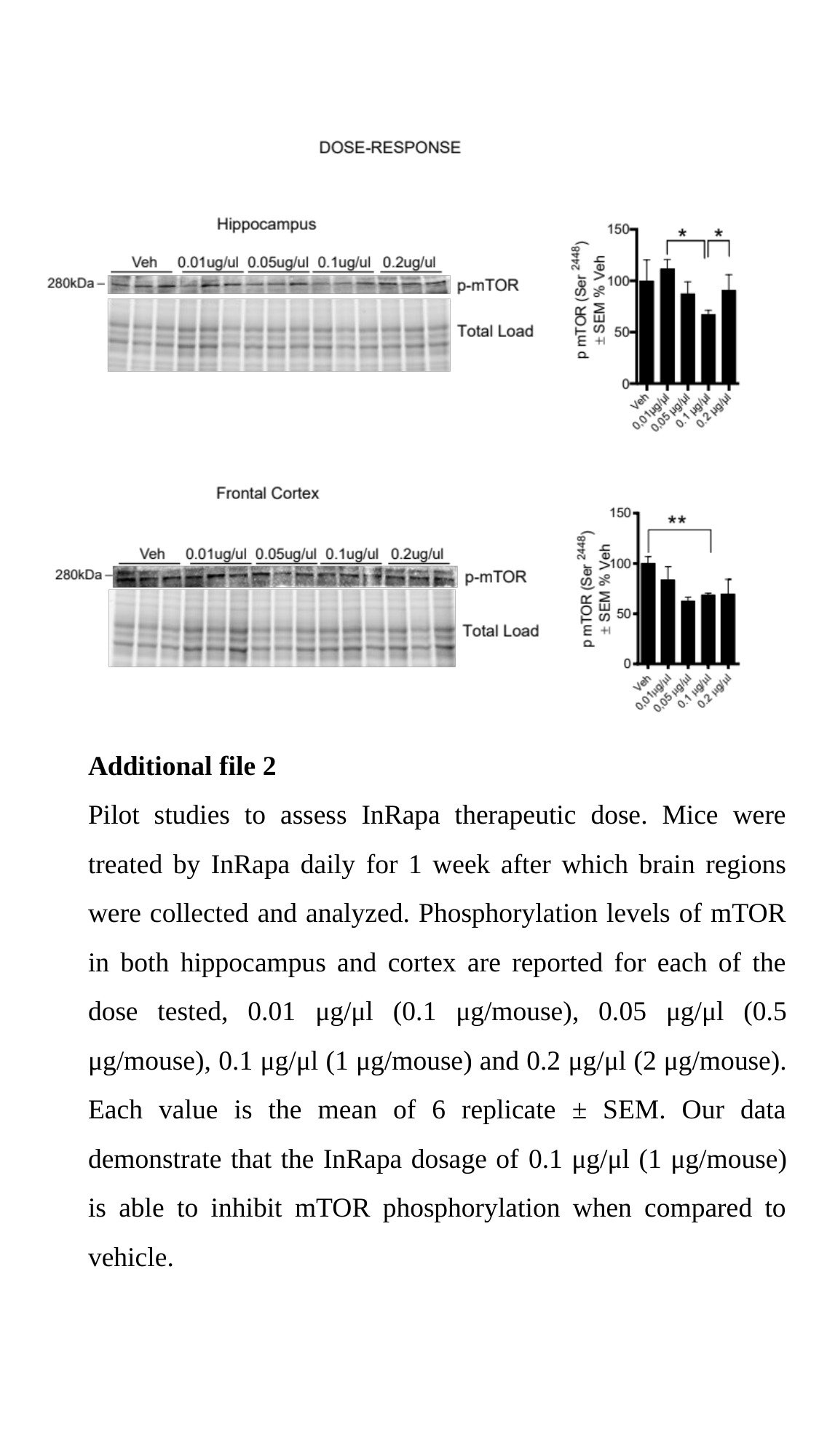

Additional file 2
Pilot studies to assess InRapa therapeutic dose. Mice were treated by InRapa daily for 1 week after which brain regions were collected and analyzed. Phosphorylation levels of mTOR in both hippocampus and cortex are reported for each of the dose tested, 0.01 μg/μl (0.1 μg/mouse), 0.05 μg/μl (0.5 μg/mouse), 0.1 μg/μl (1 μg/mouse) and 0.2 μg/μl (2 μg/mouse). Each value is the mean of 6 replicate ± SEM. Our data demonstrate that the InRapa dosage of 0.1 μg/μl (1 μg/mouse) is able to inhibit mTOR phosphorylation when compared to vehicle.
