# Supplementary material for: Intranasal rapamycin ameliorates Alzheimer-like cognitive decline in a mouse model of Down syndrome
Source: Transl Neurodegener. 2018 Nov 6;7:28. doi: 10.1186/s40035-018-0133-9 (PMC6218962; doi:10.1186/s40035-018-0133-9)
Supplement: Supplementary file 4 — Table reporting 2-way ANOVA data analysis. For 2-way ANOVA analysis only proteins showing changes before and after the InRapa treatment have been taken under consideration. (PPTX 844 kb) [file 40035_2018_133_MOESM4_ESM.pptx]

## Slide 1
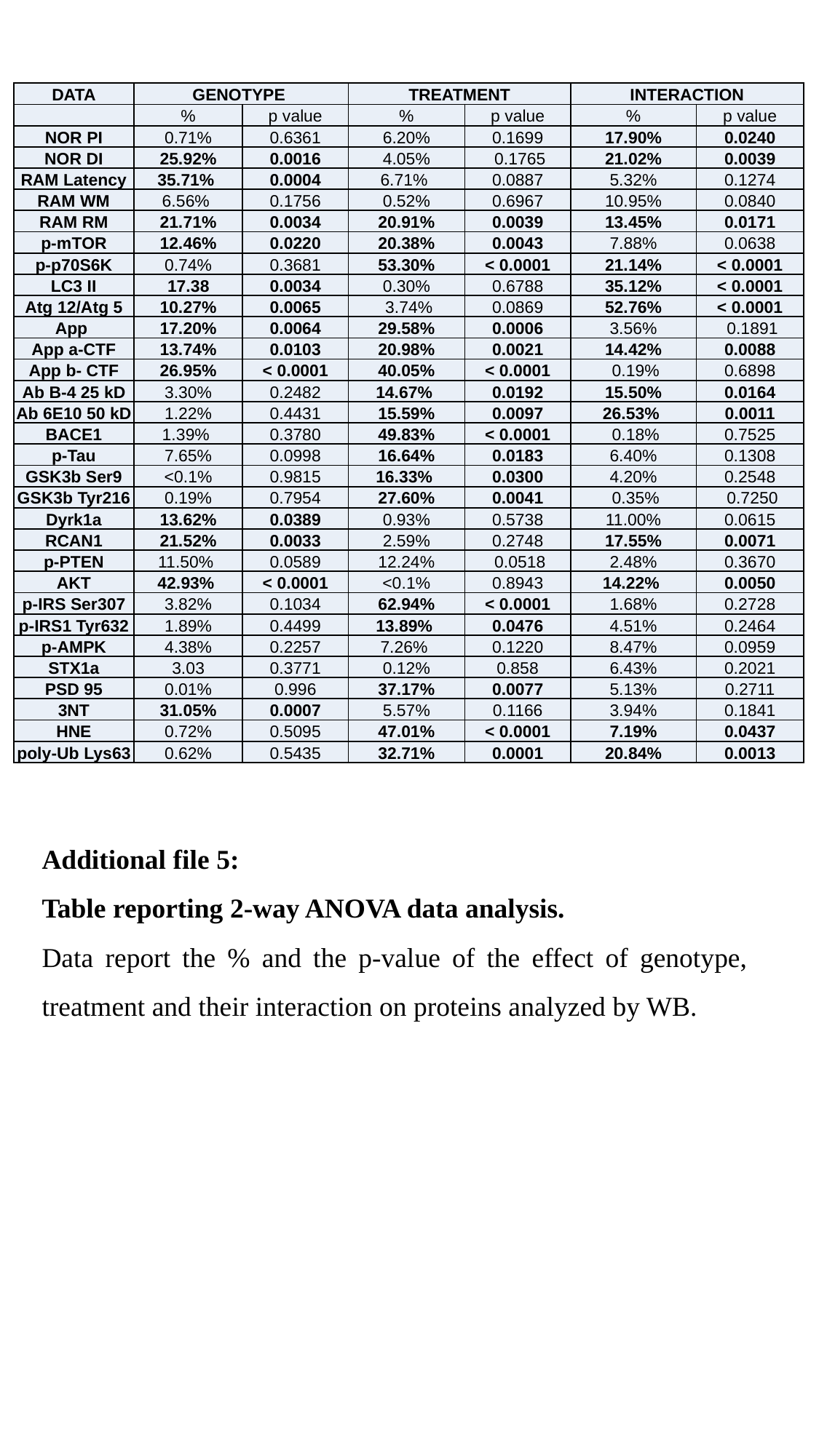

| DATA | GENOTYPE | | TREATMENT | | INTERACTION | |
| --- | --- | --- | --- | --- | --- | --- |
| | % | p value | % | p value | % | p value |
| NOR PI | 0.71% | 0.6361 | 6.20% | 0.1699 | 17.90% | 0.0240 |
| NOR DI | 25.92% | 0.0016 | 4.05% | 0.1765 | 21.02% | 0.0039 |
| RAM Latency | 35.71% | 0.0004 | 6.71% | 0.0887 | 5.32% | 0.1274 |
| RAM WM | 6.56% | 0.1756 | 0.52% | 0.6967 | 10.95% | 0.0840 |
| RAM RM | 21.71% | 0.0034 | 20.91% | 0.0039 | 13.45% | 0.0171 |
| p-mTOR | 12.46% | 0.0220 | 20.38% | 0.0043 | 7.88% | 0.0638 |
| p-p70S6K | 0.74% | 0.3681 | 53.30% | < 0.0001 | 21.14% | < 0.0001 |
| LC3 II | 17.38 | 0.0034 | 0.30% | 0.6788 | 35.12% | < 0.0001 |
| Atg 12/Atg 5 | 10.27% | 0.0065 | 3.74% | 0.0869 | 52.76% | < 0.0001 |
| App | 17.20% | 0.0064 | 29.58% | 0.0006 | 3.56% | 0.1891 |
| App a-CTF | 13.74% | 0.0103 | 20.98% | 0.0021 | 14.42% | 0.0088 |
| App b- CTF | 26.95% | < 0.0001 | 40.05% | < 0.0001 | 0.19% | 0.6898 |
| Ab B-4 25 kD | 3.30% | 0.2482 | 14.67% | 0.0192 | 15.50% | 0.0164 |
| Ab 6E10 50 kD | 1.22% | 0.4431 | 15.59% | 0.0097 | 26.53% | 0.0011 |
| BACE1 | 1.39% | 0.3780 | 49.83% | < 0.0001 | 0.18% | 0.7525 |
| p-Tau | 7.65% | 0.0998 | 16.64% | 0.0183 | 6.40% | 0.1308 |
| GSK3b Ser9 | <0.1% | 0.9815 | 16.33% | 0.0300 | 4.20% | 0.2548 |
| GSK3b Tyr216 | 0.19% | 0.7954 | 27.60% | 0.0041 | 0.35% | 0.7250 |
| Dyrk1a | 13.62% | 0.0389 | 0.93% | 0.5738 | 11.00% | 0.0615 |
| RCAN1 | 21.52% | 0.0033 | 2.59% | 0.2748 | 17.55% | 0.0071 |
| p-PTEN | 11.50% | 0.0589 | 12.24% | 0.0518 | 2.48% | 0.3670 |
| AKT | 42.93% | < 0.0001 | <0.1% | 0.8943 | 14.22% | 0.0050 |
| p-IRS Ser307 | 3.82% | 0.1034 | 62.94% | < 0.0001 | 1.68% | 0.2728 |
| p-IRS1 Tyr632 | 1.89% | 0.4499 | 13.89% | 0.0476 | 4.51% | 0.2464 |
| p-AMPK | 4.38% | 0.2257 | 7.26% | 0.1220 | 8.47% | 0.0959 |
| STX1a | 3.03 | 0.3771 | 0.12% | 0.858 | 6.43% | 0.2021 |
| PSD 95 | 0.01% | 0.996 | 37.17% | 0.0077 | 5.13% | 0.2711 |
| 3NT | 31.05% | 0.0007 | 5.57% | 0.1166 | 3.94% | 0.1841 |
| HNE | 0.72% | 0.5095 | 47.01% | < 0.0001 | 7.19% | 0.0437 |
| poly-Ub Lys63 | 0.62% | 0.5435 | 32.71% | 0.0001 | 20.84% | 0.0013 |
Additional file 5:
Table reporting 2-way ANOVA data analysis.
Data report the % and the p-value of the effect of genotype, treatment and their interaction on proteins analyzed by WB.
