# Supplementary material for: Intranasal rapamycin ameliorates Alzheimer-like cognitive decline in a mouse model of Down syndrome
Source: Transl Neurodegener. 2018 Nov 6;7:28. doi: 10.1186/s40035-018-0133-9 (PMC6218962; doi:10.1186/s40035-018-0133-9)
Supplement: Supplementary file 5 — Rapamycin distribution by UPLC-MS. Chromatograms of rapamycin in plasma (A) and brain (B) from animals treated by single I.P. injection of 50 μg/mouse (2,5 mg/kg/mouse) 4 h before sacrifice. Chromatograms of rapamycin in plasma (C) and brain (D) from animals treated by single InRapa administration of 1 μg/mouse (0.05 mg/Kg/mouse) 4 h before sacrifice. (PPTX 50 kb) [file 40035_2018_133_MOESM5_ESM.pptx]

## Slide 1
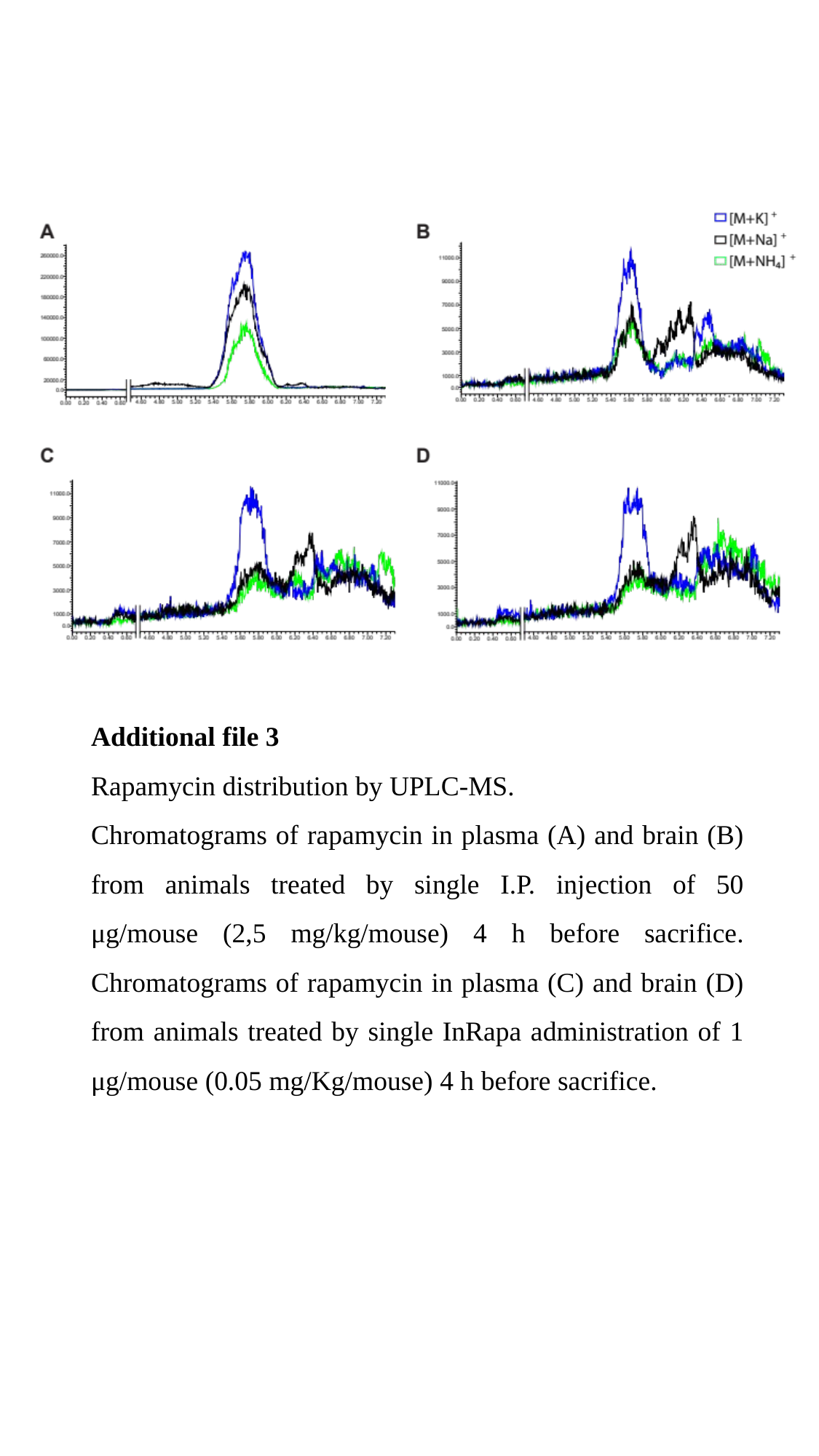

Additional file 3
Rapamycin distribution by UPLC-MS.
Chromatograms of rapamycin in plasma (A) and brain (B) from animals treated by single I.P. injection of 50 μg/mouse (2,5 mg/kg/mouse) 4 h before sacrifice. Chromatograms of rapamycin in plasma (C) and brain (D) from animals treated by single InRapa administration of 1 μg/mouse (0.05 mg/Kg/mouse) 4 h before sacrifice.
