# Supplementary material for: Intranasal rapamycin ameliorates Alzheimer-like cognitive decline in a mouse model of Down syndrome
Source: Transl Neurodegener. 2018 Nov 6;7:28. doi: 10.1186/s40035-018-0133-9 (PMC6218962; doi:10.1186/s40035-018-0133-9)
Supplement: Supplementary file 6 — Western blot analysis of mTOR and p70S6K phosphorylation in liver and heart tissue after InRapa treatment. Graph bars are reported as percentage in respect to euploid vehicle group, which is set as 100%. Data Show no significant alteration in Ts65Dn undergoing rapamycin (black bar) or vehicle (checquered bars) after intranasal delivery supporting no effects of InRapa treatment at peripheral level. (PPTX 72 kb) [file 40035_2018_133_MOESM6_ESM.pptx]

## Slide 1
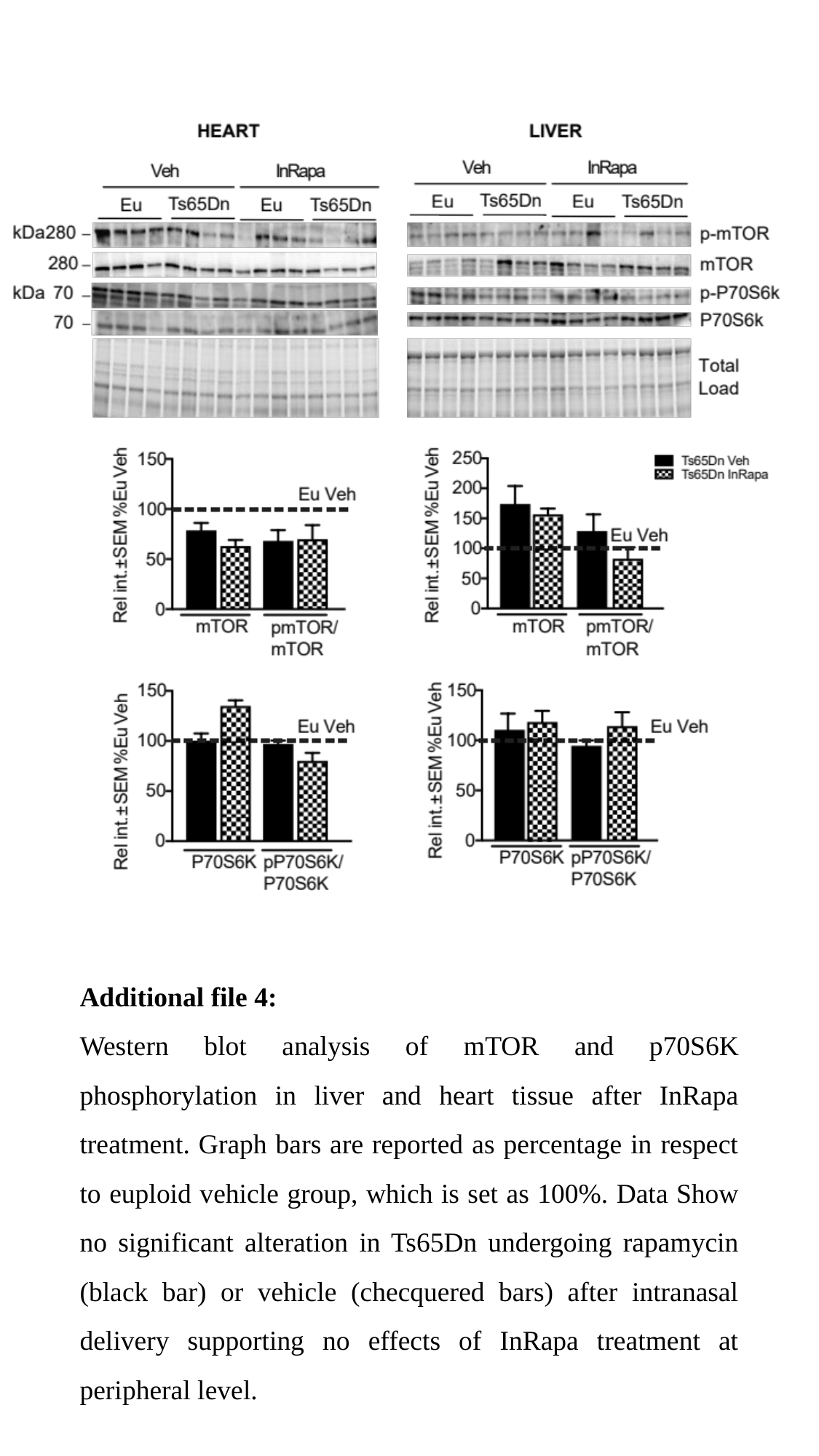

Additional file 4:
Western blot analysis of mTOR and p70S6K phosphorylation in liver and heart tissue after InRapa treatment. Graph bars are reported as percentage in respect to euploid vehicle group, which is set as 100%. Data Show no significant alteration in Ts65Dn undergoing rapamycin (black bar) or vehicle (checquered bars) after intranasal delivery supporting no effects of InRapa treatment at peripheral level.
