# Supplementary material for: Intranasal rapamycin ameliorates Alzheimer-like cognitive decline in a mouse model of Down syndrome
Source: Transl Neurodegener. 2018 Nov 6;7:28. doi: 10.1186/s40035-018-0133-9 (PMC6218962; doi:10.1186/s40035-018-0133-9)
Supplement: Supplementary file 7 — Immunofluorescence staining of Dentate gyrus in Eu and Ts65Dn mice. Representative immunofluorescent images showing (A) p-mTOR at serine 2448, (B) at Ser416 and (C) APP/Ab levels in the dentate gyrus region of the hippocampus from euploid mice treated with Veh and InRapa (A.1–4), and Ts65Dn mice treated with Veh and InRapa (A.5–8). DAPI (blue) was used to identify cell nuclei. Scale bar represent 20 μm. On the right of each panel a graph of the quantification of fluorescence signal is reported. (PPTX 16410 kb) [file 40035_2018_133_MOESM7_ESM.pptx]

## Slide 1
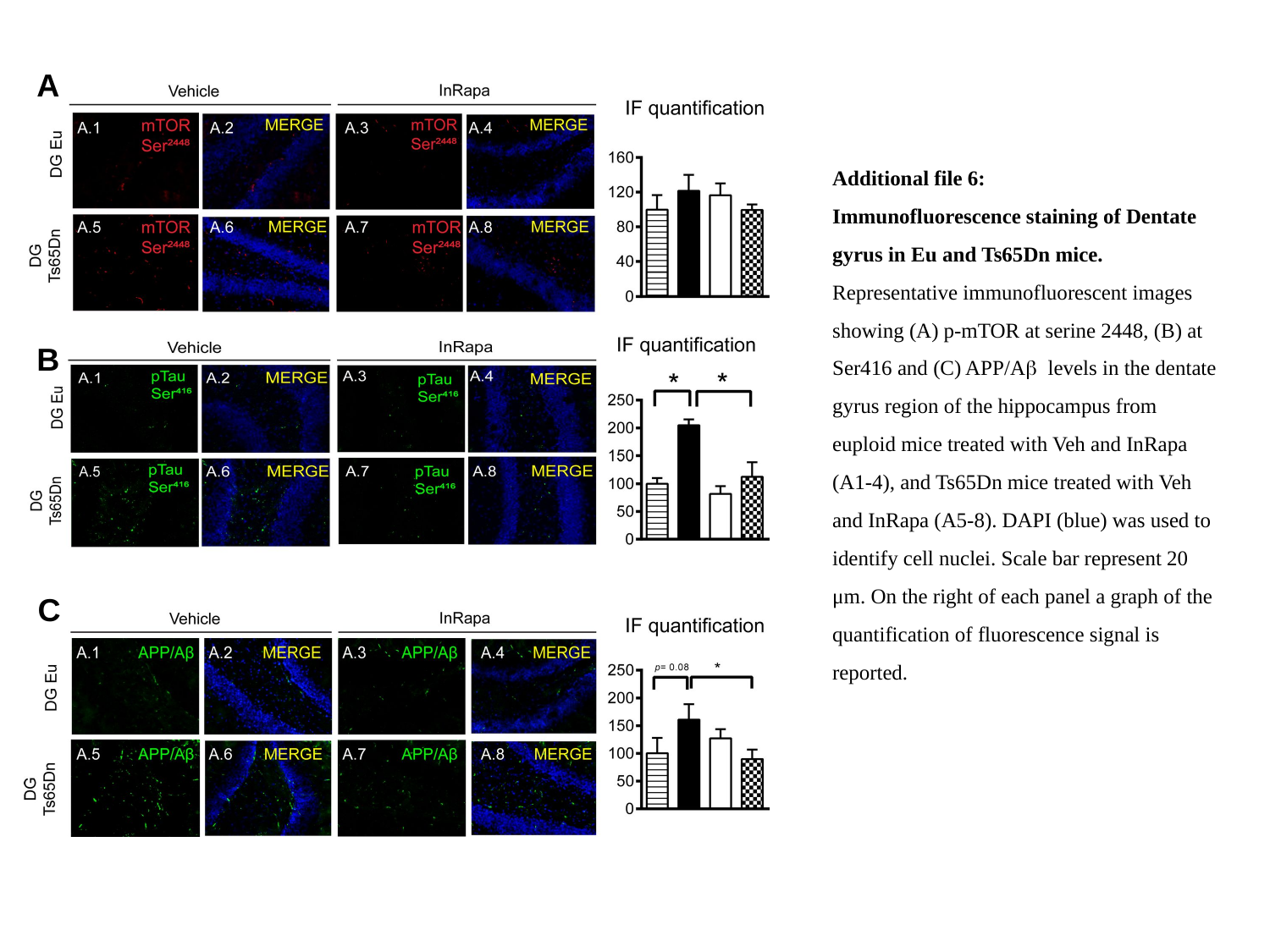

A
Additional file 6:
Immunofluorescence staining of Dentate gyrus in Eu and Ts65Dn mice.
Representative immunofluorescent images showing (A) p-mTOR at serine 2448, (B) at Ser416 and (C) APP/Ab levels in the dentate gyrus region of the hippocampus from euploid mice treated with Veh and InRapa (A1-4), and Ts65Dn mice treated with Veh and InRapa (A5-8). DAPI (blue) was used to identify cell nuclei. Scale bar represent 20 μm. On the right of each panel a graph of the quantification of fluorescence signal is reported.
B
C
